# Comparative efficacy of 10 Chinese herbal injections combined with GP regimen chemotherapy for patients with advanced NSCLC a systematic review and network meta-analysis

**DOI:** 10.7150/jca.66410

**Published:** 2022-01-01

**Authors:** Juan Li, Guang-Hui Zhu, Tong-Tong Liu, Bo-Wen Xu, Jie Li

**Affiliations:** 1 Department of Oncology, Guang' anmen Hospital, China Academy of Chinese Medical Sciences.; 2 School of Graduates, Beijing University of Chinese Medicine, Beijing.

**Keywords:** Chinese herb injections, NSCLC, GP regimen chemotherapy, network meta-analysis

## Abstract

**Background:** Numerous studies have indicated that some Chinese herbal injections (CHIs) might have a beneficial treatment effect when used in combination with chemotherapy. However, the results of these studies have been inconsistent. The aim of this network meta-analysis (NMA) was to evaluate and compare the clinical efficacy and safety of different CHIs combined with gemcitabine plus cisplatin (GP) regimen chemotherapy with that of GP regimen chemotherapy alone in the treatment of patients with advanced non-small cell lung cancer (NSCLC).

**Materials and Methods:** Eight databases were systematically searched to identify randomized clinical trials (RCTs) from the date of inception of the database to August 11, 2021. The primary outcome measures were the objective response rate (ORR) and adverse reactions (including nausea and vomiting, and leukopenia). The secondary outcome measures were median survival time (MST) and quality of life (QOL). The quality of the included studies was assessed using the Cochrane risk of bias tool. Standard pair-wise and Bayesian NMAs were carried out to compare the effectiveness and safety of different CHIs combined with GP regimen chemotherapy using WinBUGS 14 and Stata 15.1 software. Sensitivity analysis and Egger's test were also performed to check robust.

**Results:** A total of 92 eligible RCTs involving 7,728 patients and 10 CHIs were included. The results showed that Kangai injection (KAI), Kanglaite injection (KLT), Aidi injection and Compound Kushen (CKSI) injection displayed obvious advantages in both efficacy and safety. Aidi+GP (79.0%) showed great advantages of ORR, and KAI+GP and KLT+GP had the lowest probability in terms of leukopenia (4.4%) and nausea and vomiting (24.2%). Besides, KLT+GP was shown to positively affect MST. According to the subgroup analyses, CHIs might have a limited effect in reducing adverse reactions, and have a similar effect in squamous cell carcinoma and adenocarcinoma.

**Conclusions:** KAI+GP of adjuvant drugs, Aidi+GP and CKSI+GP of anticancer drugs appeared to be the advantageous treatment options for patients with advanced NSCLC, owing to its superior therapeutic performance and reduced adverse reactions. KLT+GP might prolong survival. Nevertheless, additional results from multicenter trials and high-quality studies will be pivotal in supporting our findings.

## Introduction

Lung cancer is a leading cause of malignancy-related mortality worldwide and has a high morbidity rate that is continuing to rise. Approximately 85% of lung cancers are non-small cell lung cancer (NSCLC) 1. In almost 75% of NSCLC cases, the patient presents with advanced local invasion and metastasis during hospital admission diagnosis 2, putting them beyond the stage at which surgical intervention can be applied. Recently, molecular targeted therapy and immunotherapy have emerged as therapeutic options; however, chemotherapy remains the cornerstone of NSCLC treatment 3, especially for patients with advanced (stage III/IV) disease. Gemcitabine combined with cisplatin (GP) regimen is one of the two standard platinum-based chemotherapy drug regimens. Previous research has shown that the overall survival of patients receiving GP regimen chemotherapy is noninferior to that of patients treated with cisplatin/pemetrexed. Moreover, in patients with squamous cell histology, the GP regimen showed a significant improvement in survival compared with cisplatin/pemetrexed (n = 473; 10.8 *vs.* 9.4 months, respectively) 4. However, patients who receive GP chemotherapy may experience gastrointestinal reactions, blood toxicity, and other toxic side effects, which affects their quality of life (QOL) and hinders treatment 5,6.

To increase the therapeutic effect and reduce adverse reactions in cancer treatment, drug combinations are often used. Chinese herbal injections (CHIs) are guided by the theoretical system of syndrome differentiation in traditional Chinese medicine (TCM), combined with the purification of modern advanced technology. In the basic theory of TCM, the pathogenesis of lung cancer involves Qi stagnation, blood stasis, phlegm accumulation, and cancer toxins. In China, CHIs have been used as a complementary or alternative therapy to chemotherapy in the treatment of NSCLC 7,8, particularly due to their effects of removing phlegm, blood stasis, and resolving hard lumps. However, to date, no direct head-to-head comparative evidence regarding the optimal CHI plus GP regimen chemotherapy for NSCLC treatment has been reported; thus, a network meta-analysis (NMA) that aims to compare CHIs is required.

This study classified 11 national standard CHIs according to their indications for the treatment of tumors and lung cancer 9, and 2 CHIs commonly used in NSCLC 10,11. Finally, 10 CHIs were selected: Aidi injection (Aidi), Compound Kushen injection (CKSI), Kanglaite injection (KLT), Kangai injection (KAI), Brucea javanica Oil Emulsion injection (BJOE), Shenqi Fuzheng injection (SQFZ), Xiaoaiping injection (XAPI), Astragalus polysaccharide injection (API), Lentinan injection (LTNI) and Elemene injection (ELMI). The primary aim of this study was to investigate and rank the treatment efficacy and safety of the aforementioned CHIs by performing an NMA.

## Methods

This study is presented according to the Preferred Reporting Items for Systematic Reviews and Meta-Analyses (PRISMA) guidelines “NMA extended version” 12, and the study is registered with PROSPERO (CRD42020167142). As the materials used in this study had been published previously, ethical approval was not required.

### Data sources

A literature search was performed to identify randomized clinical trials (RCTs) of CHI-assisted treatment of advanced NSCLC. Eight databases were searched including PubMed, the Cochrane Library, EMBASE, Web of Science (ISI), Chinese National Knowledge Infrastructure (CNKI), Chinese Scientific Journals Full-Text Database (VIP), CBM, and Wanfang Data. The literature search was performed by two independent reviewers (Juan Li and Guang-Hui Zhu). Searches were restricted to original publications from the date of establishment of the database to August 11, 2021. A combination of the following keywords was used: “lung cancer”, “lung carcinoma”, “non-small cell lung cancer”, “NSCLC”, “gemcitabine”, or “cisplatin”, as well as search terms for each of the CHIs. All retrievals were implemented using the Medical Subject Headings (MeSH) and free word. Besides, all related systematic reviews (SRs) and meta-analyses were evaluated, and studies meeting the inclusion criteria were selected from the references. As an example, the electronic strategy for PubMed is shown in Supplementary Figure 1.

### Search strategies and selection criteria

Studies were selected according to the following inclusion criteria: (1) The study was a RCT. (2) The patients were diagnosed with stage III and IV NSCLC according to histopathological and cytological diagnostic criteria, with the tumor-node-metastasis (TNM) classification based on the American Joint Committee on Cancer staging system 13. (3) The control group received GP regimen chemotherapy alone, while the experimental group was treated with GP regimen chemotherapy combined with CHIs. The CHI therapeutic interventions included the following 13 intravenous CHIs: Aidi, Toad Venom Injection (TVI), CKSI, Huachansu injection (HCSI), KLT, KAI, BJOE, SQFZ, XAPI, API, LTNI, ELMI and Ginseng Polysaccharide Injection (GPI). (4) Patients had not received any radiotherapy, other chemotherapy, or other Chinese herbs during the study. (5) The study outcome needed to at least include an objective response rate (ORR) or adverse reactions (nausea and vomiting, and leukopenia).

Duplicates, non-RCTs (including case-control studies and series case reports), unrelated studies (including those on other treatments), abstracts and reviews without specific data, unrelated SRs, and studies with no information regarding the pharmaceutical company or drug number of the CHI used were all excluded.

### Data extraction and quality assessment

Two researchers (Guang-Hui Zhu and Bo-wen Xu) independently extracted the following information from each study: basic information (lead author, publication date, demographic characteristics, and sample size), characteristics of the intervention (type and usage of CHIs, treatment duration, evaluation criteria of clinical efficacy, and supportive treatments such as anti-nausea drugs and granulocyte colony-stimulating factor), and outcomes. The risk of bias of the included studies was assessed by two authors (Juan Li and Tong-Tong Liu) using the RCT bias risk assessment tool recommended by the Cochrane risk-of-bias criteria 14, and cross-checked finally. Any disagreements were resolved by a third reviewer (Jie Li).

### Main outcomes

Tumor response was assessed according to the ORR. According to the World Health Organization (WHO) guidelines for solid tumor responses 15, or the Response Evaluation Criteria in Solid Tumors (RECIST) 16. Tumors were evaluated as complete response (CR), partial response (PR), stable disease (SD), or progressive disease (PD), with the ORR being equal to CR plus PR. Adverse reactions (adverse drug events or adverse drug reactions) were pooled, including nausea and vomiting, and leukopenia.

### Secondary outcomes

Median survival time (MST) was considered to represent the long-term synergistic efficacy of a combination therapy. Furthermore, the secondary outcomes also included QOL, which was considered to be improved if a patient's Karnofsky Performance status (KPS) score increased by 10 points or more following treatment 17.

### Statistical analysis

The Bayesian NMA was performed using WinBUGS 14 and Stata 15.1 software. Stata software was applied to draw the network evidence map of the NMA, as well as to test the inconsistency (based on the closed loops and node-splitting model). If the difference exhibited statistical significance (*P* < 0.05), the consistency model was used for analysis and the results were sorted. Otherwise, the inconsistency model was used. The odds ratios (ORs) and 95% confidence intervals (CIs) of the dichotomous variables were used as the effect-quantity indexes. For survival outcomes, MST was presented as a hazard ratio (HR) with 95% CI. Pairwise meta-analysis was conducted according to heterogeneity. A frequentist framework, random-effects NMA was used to compare all classes of CHIs for each pre-specified outcome. The surface under the cumulative ranking curves (SUCRA) was used to assess the efficacy of each drug intervention program; interventions with higher SUCRA values were associated with the highest probability of being more effective. The comparison-adjusted funnel plot was completed to evaluate the clinical and methodological heterogeneity. Subgroup analyses were performed following the cycle of chemotherapy, supportive treatment, evaluation criteria, and pathological types of NSCLC to demonstrate the clinical heterogeneity and its influence on the endpoint.

## Results

### Study selection and characteristics

**Figure 1:**
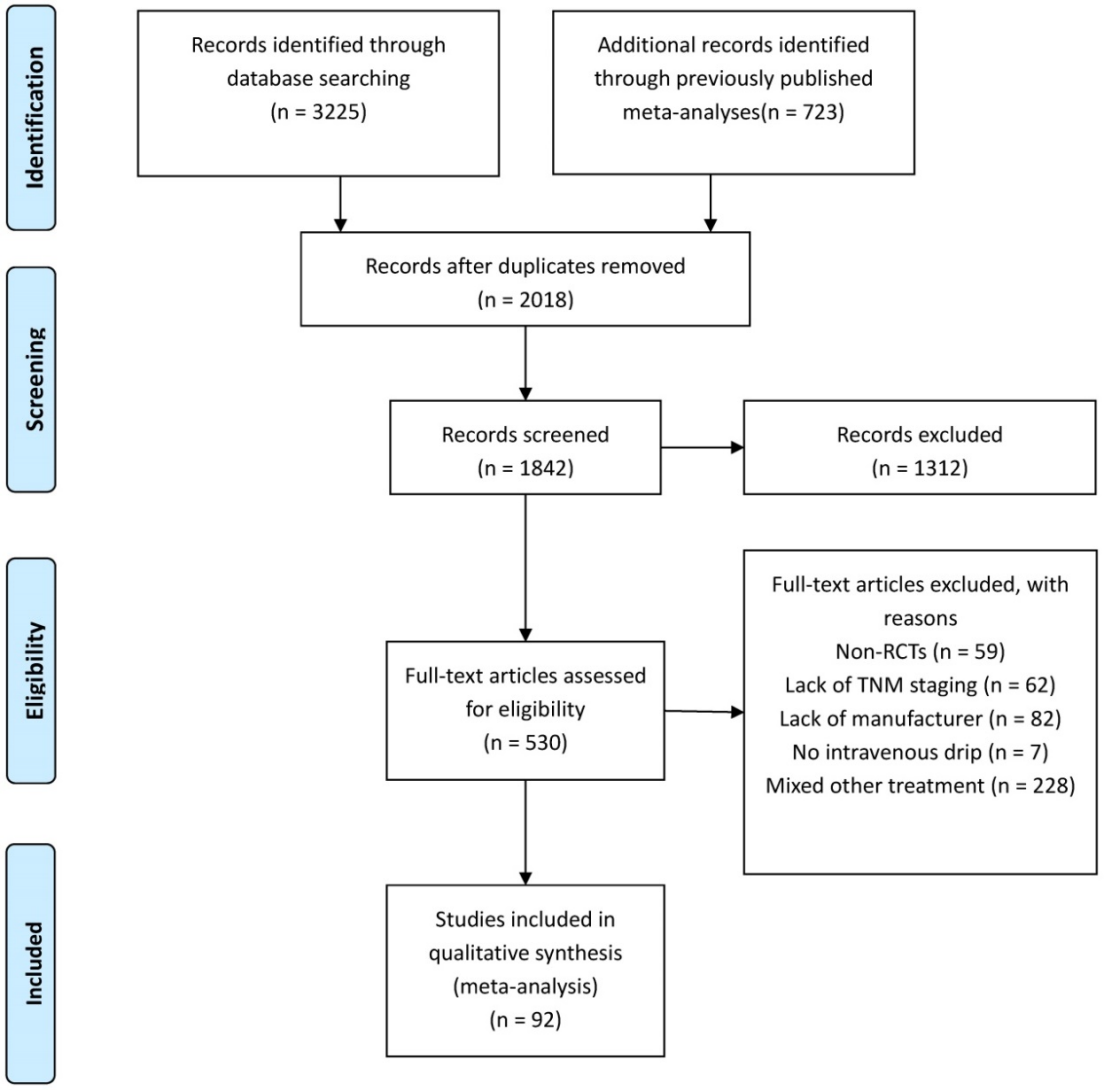
Articles retrieved and assessed for eligibility.

A total of 3,957 relevant studies were identified through systematic searching and previously published meta-analyses. After the removal of duplicates, leaving 1,842 potentially eligible records. A further 1,312 reviews, case reports, and unrelated studies were excluded after the titles and abstracts were read. Next, the full texts of the remaining 530 studies were assessed for eligibility. There were 438 full-text articles that did not meet the inclusion and exclusion criteria, such as Non-RCTs (n = 59), Lack of TNM staging (n = 62), Lack of manufacturer (n = 82), No intravenous drip (n = 7), Mixed other treatment (n = 228) (Figure 1). Finally, 92 studies 18-109 involving 7,728 patients were finally included in this NMA to compare the efficacy and safety of 10 CHIs (Aidi, KLT, CKSI, KAI, BJOE, SQFZ, XAPI, API, LTNI, and ELMI) plus GP regimen chemotherapy. All of the included studies were conducted in China (Table 1).

**Figure 2:**
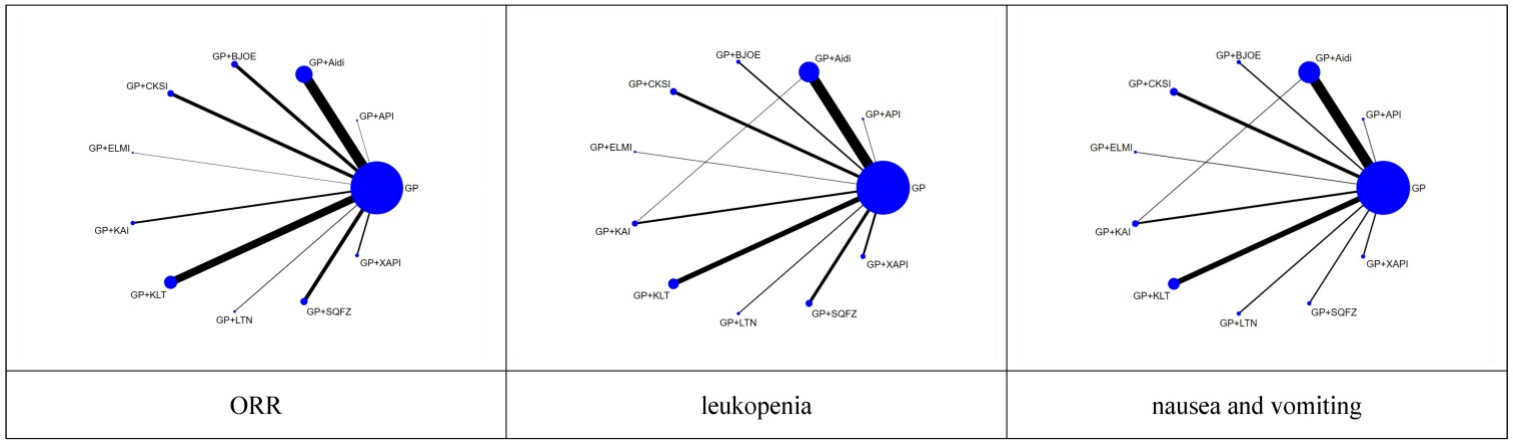
Network graph of the main outcomes. **NOTE:** Node sizes indicate the total sample sizes for treatments, and the line thickness corresponds to the number of trials.

For each of the CHIs, the number of included studies was as follows: Aidi, 28 trials; KLT, 20 trials; CKSI, 9 trials; KAI, 6 trials; BJOE, 10 trials; SQFZ, 10 trials; XAPI, 4 trials; API, 1 trial; LTNI, 2 trials; ELMI, 1 trial and 1 trial includes Aidi and KAI 27. A network plot of the main outcomes of the Bayesian analysis is depicted in Figure 2. And the Node-splitting model resylts for main outcomes showed no difference presented statistically significance (*P* > 0.05), the consistency model was used for analysis (Supplementary Table 2).

**Figure 3:**
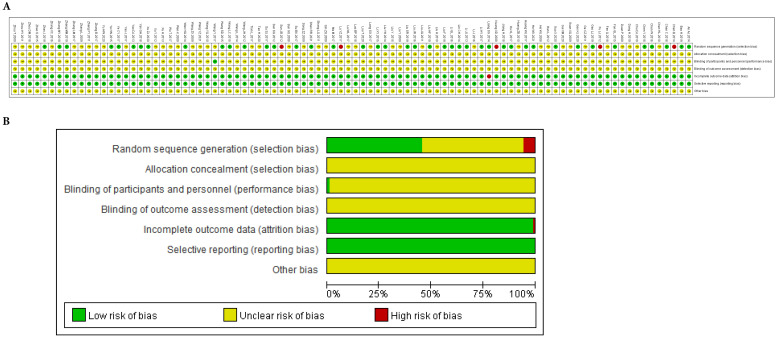
** Risk of methodological bias. A.** Risk of bias summary: review authors' judgments about each risk of bias item for each included study. **B.** Risk of bias graph: review authors' judgements about each risk of bias item presented as percentages across all included studies.

There were 41 studies 20-22, 26-30, 32, 35-36, 41, 44, 47, 49-50, 54-56, 60, 63, 65-66, 68, 70, 74, 77-78, 80-81, 83, 86, 88-90, 93, 98, 102, 104, 108 that adopted a random number table, and 2 studies 48, 103 that used direct sampling, to randomize subjects into groups. These studies were rated as having a low risk of bias. Five studies 18-19, 23, 34, 99 were based on the patient's opinion or medical record number, which were rated as having a high risk of bias. None of the included studies reported the details of concealed allocations. One study 62 involved the blinding of patients. Five patients who presented with acute/subacute toxicity withdrew from one study 59. Besides, in 10 of the studies 33, 49, 51, 54-55, 62, 76, 82, 85, doses were unknown or not recommended. The methodological bias risk of all included studies is presented in Figure 3 and Supplementary Table 1.

### Objective response rate (ORR)

**Figure 4:**
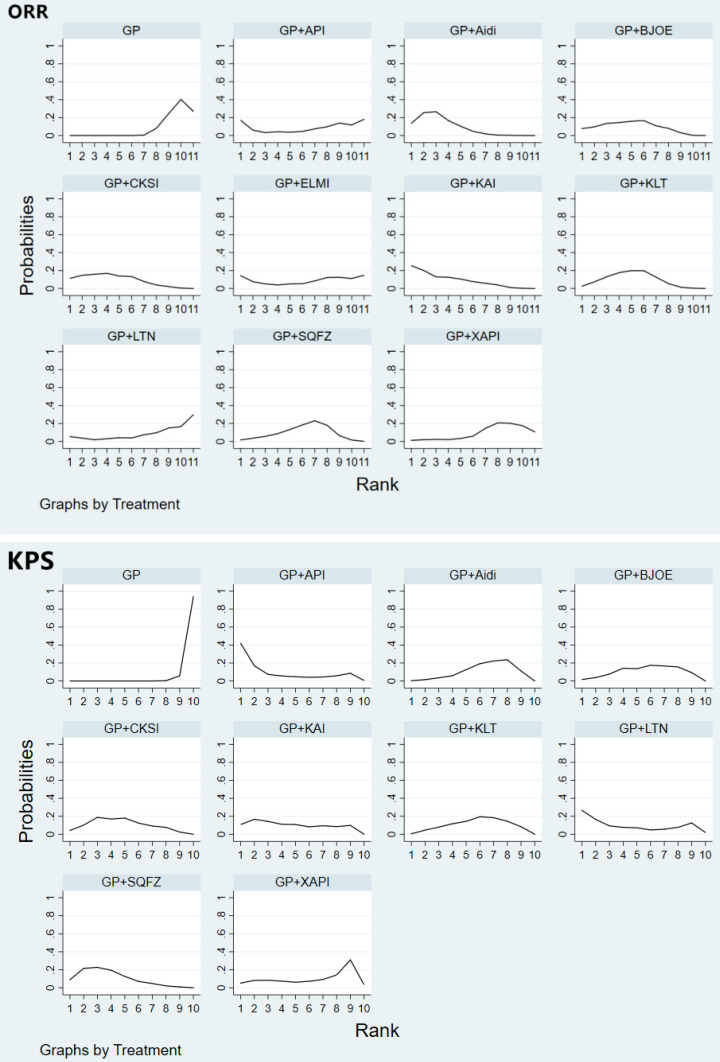
Rank of the cumulative probabilities for the ORR and KPS. **NOTE:** Higher surface under the cumulative ranking curves (SUCRA) values indicated higher probabilities that the treatments were more effective and superior than other therapies.

ORR was reported in 88 studies involving 10 CHIs. The results revealed that, compared with the effects of GP regimen chemotherapy alone, GP+Aidi (OR = 1.87, 95%CI [1.56, 2.24]), GP+KLT (OR = 1.66, 95%CI [1.35, 2.04]), GP+CKSI (OR = 1.76, 95%CI [1.33, 2.33]), GP+BJOE (OR = 1.70, 95%CI [1.28, 2.27]), GP+SQFZ (OR = 1.52, 95%CI [1.16, 2.01]), and GP+KAI (OR = 1.88, 95%CI [1.33, 2.65]) (*P* < 0.05) were associated with a significantly improved ORR (Table 2, Supplementary Figure 2). According to the SUCRA analysis of the ORR, the 10 CHIs were ranked as follows: GP+Aidi (79.0%) > GP+KAI (76.4%) > GP+CKSI (68.6%) > GP+BJOE (62.6%) > GP+KLT (60.8%) > GP+SQFZ (48.5%) > GP+ELMI (44.2%) > GP+API (42.5%) > GP+XAPI (28.8%) > GP+LTNI (27.1%) > GP (11.5%) (Figure 4).

### Adverse reactions

**Figure 5:**
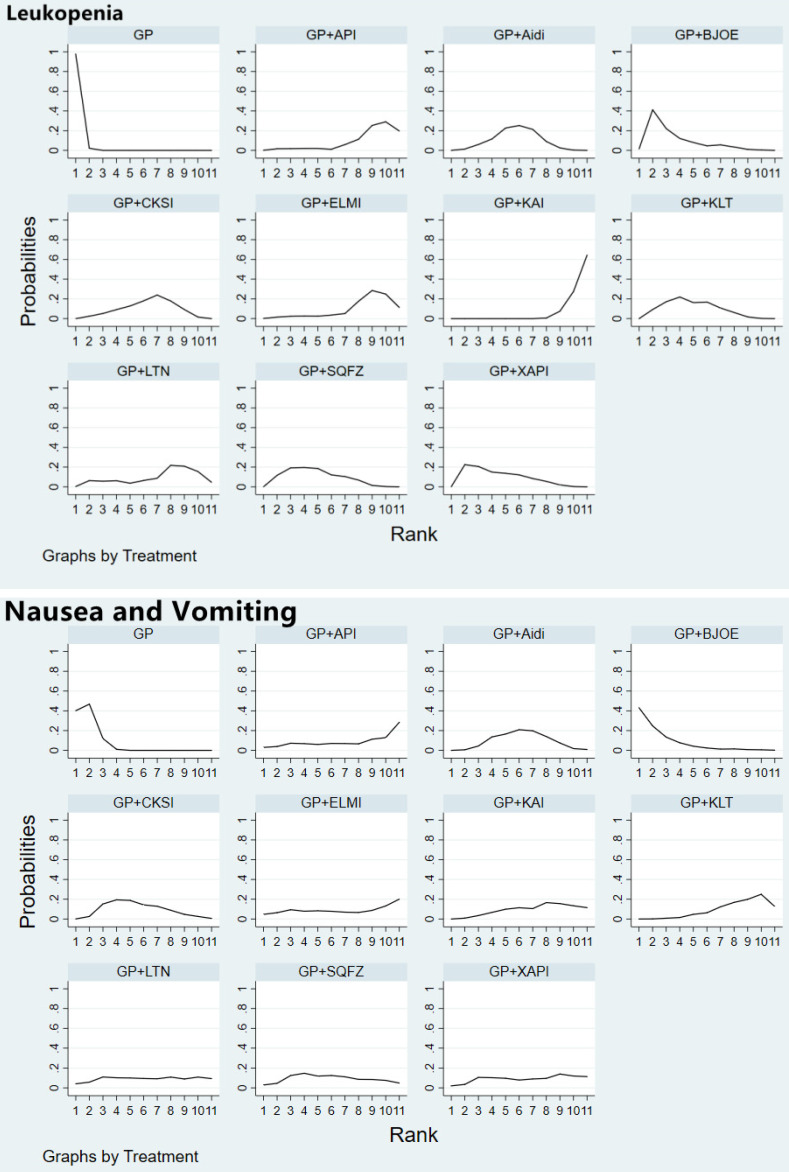
Rank of the cumulative probabilities for the leukopenia, nausea and vomiting. **NOTE:** Higher surface under the cumulative ranking curves (SUCRA) values indicated higher probabilities that the treatments had more adverse reactions than other therapies.

Fifty-six studies involving 10 CHIs reported on leukopenia. The results showed that GP+Aidi (OR = 0.40, 95%CI [0.30, 0.52]), GP+KLT (OR = 0.44, 95%CI [0.31, 0.64]), GP+CKSI (OR = 0.37, 95%CI [0.34, 0.55]), GP+BJOE (OR = 0.55, 95%CI [0.31, 0.97]), GP+SQFZ (OR = 0.45, 95%CI [0.31, 0.67]), GP+KAI (OR = 0.13, 95%CI [0.07, 0.23]), GP+XAPI (OR = 0.48, 95%CI [0.29, 0.79]), GP+API (OR = 0.20, 95%CI [0.07, 0.59]), GP+LTNI (OR = 0.29, 95%CI [0.11, 0.75]) and GP+ELMI (OR = 0.23, 95%CI [0.09, 0.59]) (*P* < 0.05) were associated with lower rates of leukopenia than GP regimen chemotherapy alone. Besides, GP+KAI carried a lower risk of leukopenia than GP+SQFZ (OR = 0.28, 95%CI [0.14, 0.56]), GP+BJOE (OR = 0.23, 95%CI [0.10, 0.52]), GP+CKSI (OR = 0.34, 95%CI [0.17, 0.69]), GP+KLT (OR = 0.28, 95%CI [0.14, 0.56]), and GP+Aidi (OR = 0.31, 95%CI [0.17, 0.60]) (*P* < 0.05). GP+XAPI was observed to have a higher risk of leukopenia than GP+KAI (OR = 3.82, 95%CI [1.76, 8.28]) (*P* < 0.05) (Table 3, Supplementary Figure 3). Based on the SUCRA analysis of different schemes intervene leukopenia, GP+KAI may be a lower risk treatment option for endpoint events. In terms of the risk of endpoint events, the treatment options were ranked as follows: GP+KAI (4.4%) < GP+API (20.0%) < GP+ELMI (24.0%) < GP+LTNI (36.0%) < GP+CKSI (46.3%) < GP+Aidi (52.2%) < GP+KLT (61.9%) < GP+SQFZ (63.2%) < GP+XAPI (67.0%) < GP+BJOE (75.3%) < GP (99.8%) (Figure 5).

Forty studies involving 10 CHIs reported that the GP regimen involved a higher risk of nausea and vomiting than GP+Aidi (OR = 2.09, 95%CI [1.54, 2.83]), GP+KLT (OR = 2.82, 95%CI [1.74, 4.48]), GP+CKSI (OR = 1.87, 95%CI [1.13, 3.09]) and GP+KAI (OR = 2.50, 95%CI [1.29, 4.84]) (*P* < 0.05). Furthermore, GP+BJOE carried a higher risk than GP+KLT (OR = 0.37, 95%CI [0.15, 0.99]) (*P* < 0.05) (Table 3, Supplementary Figure 4). In terms of the risk of nausea and vomiting as an adverse effect, the CHIs were ranked as follows: GP+KLT (24.2%) < GP+API (31.3%) < GP+KAI (35.8%) < GP+ELMI (39.0%) < GP+XAPI (41.0%) < GP+LTNI (45.5%) < GP+Aidi (48.3%) < GP+SQFZ (49.5%) < GP+CKSI (56.9%) < GP+BJOE (85.4%) < GP (93.1%) (Figure 5).

### Effects on secondary outcomes

For secondary outcomes, 11 studies 18, 21, 32, 42, 49, 54, 64, 73, 96-97, 104 reported the MST, involving Aidi, KLT, XAPI, CKSI and ELMI (Supplementary Figure 5). The NMA demonstrated that, when compared with the GP regimen alone, GP+KLT (HR = 1.45, 95%CI [1.12, 1.86]) (*P* < 0.05) was the only combination that showed a statistical difference. Based on the probabilistic collation table, GP+KLT performed best with regard to MST (Supplementary Figure 6).

KPS score was reported in 37 studies involving 9 CHIs (Supplementary Figure 5). The results revealed that GP regimen chemotherapy was associated with lower KPS scores than GP+Aidi (OR = 0.40, 95%CI [0.29, 0.56]), GP+KLT (OR = 0.38, 95%CI [0.26, 0.56]), GP+CKSI (OR = 0.32, 95%CI [0.21, 0.49]), GP+BJOE (OR = 0.38, 95%CI [0.24, 0.58]), GP+SQFZ (OR = 0.29, 95%CI [0.19, 0.44]), GP+KAI (OR = 0.33, 95%CI [0.15, 0.71]), GP+API (OR = 0.24, 95%CI [0.07, 0.78]) and GP+LTNI (OR = 0.29, 95%CI [0.09, 0.94]) (*P* < 0.05) (Table 2). According to the SUCRA of KPS, the 9 CHIs were ranked as follows: GP+API (74.6%) > GP+SQFZ (71.0%) > GP+LTNI (64.7%) > GP+KAI (60.0%) > GP+CKSI (59.8%) > GP+BJOE (45.8%) > GP+KLT (45.6%) > GP+XAPI (40.0%) > GP+Aidi (37.7%) > GP (0.7%) (Figure 4). The SUCRAs of the different treatments outcomes are shown in Table 4.

### Cluster analysis

We used cluster analysis to comprehensively compare the effects of the interventions on two different outcomes. The first cluster analysis was performed for 9 types of CHIs for which ORR and KPS were reported. The plot was based on the SUCRA values of the CHI groups; each color represents a set of treatment groups that belong to the same cluster, and treatment groups that were located in the upper right corner were superior to other CHIs in both the ORR and KPS. The results of the cluster analysis demonstrated that GP+Aidi, GP+KAI and GP+CKSI had better therapeutic effects than others. Besides, three cluster analyses were conducted for 10 types of CHIs. GP+KAI had advantages in the ranking of ORR and leukopenia and was found to be a tolerable option for the relief of ADRs. As for ORR and nausea, KLT+GP showed better advantages in ranking.

**Figure 6:**
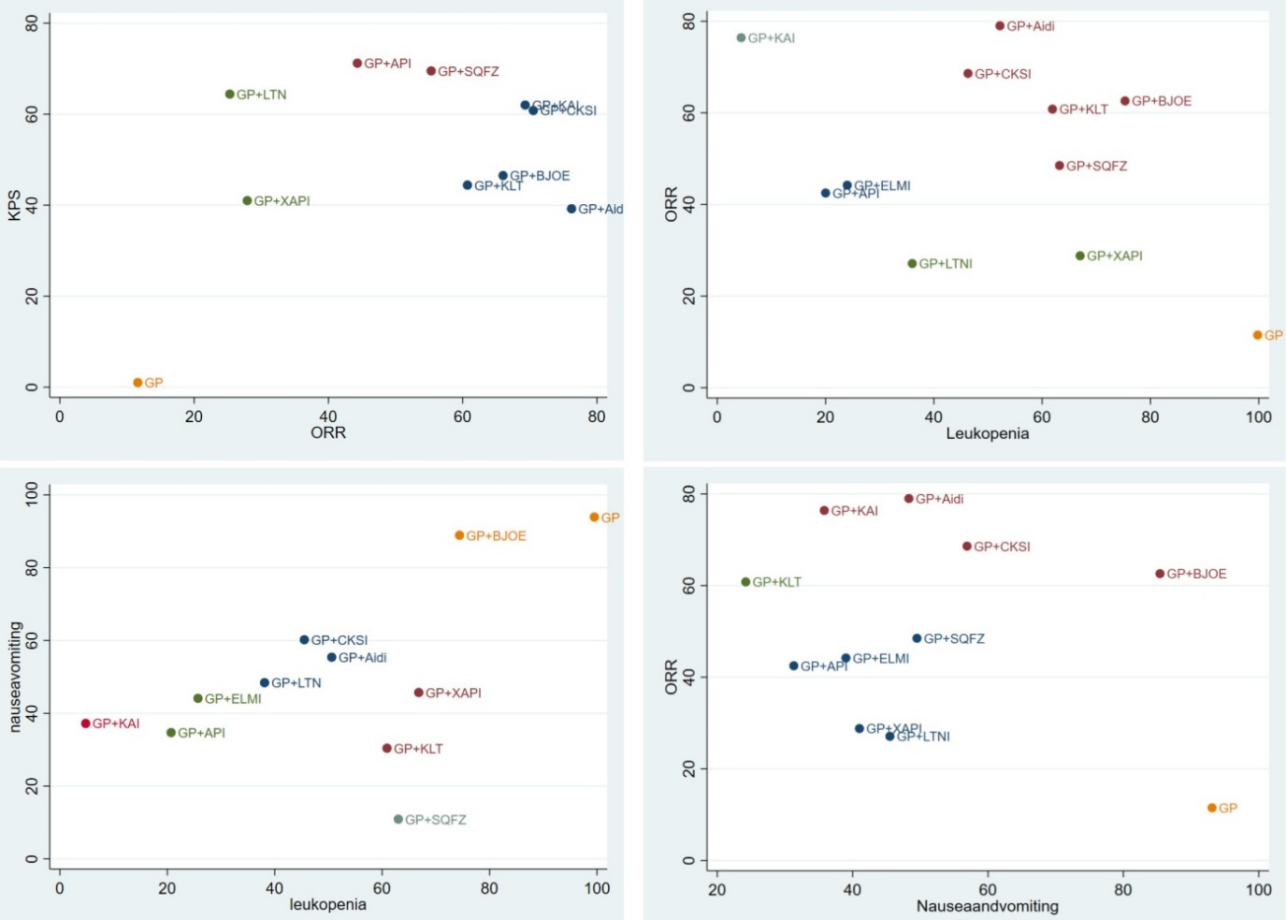
Cluster analysis plots.** NOTE:** Interventions in the upper-right section of the cluster analysis plots were more easy Leading to relevant results. GP: cisplatin and gemcitabine; Aidi: Aidi injection; KLT: Kanglaite injection; CKSI: Compound Kushen injection; BJOE: Brucea javanica Oil Emulsion injection; SQFZ: Shenqi Fuzheng injection; KAI: Kangai injection; XAPI: Xiaoaiping injection; API: Astragalus polysaccharide injection.

Moreover, we paid more attention to anticancer drugs (ZC01) included Aidi, CKSI, XAIP, BJOE from the catalog of drugs for national basic medical insurance of China 18. Aidi and CKSI showed better advantages in ranking of efficient and safety in this study. In contrast, GP regimen chemotherapy alone was comprehensively ranked as having the worst efficacy and safety of the treatment options (Figure 6).

### Subgroup and sensitivity analyses

The included studies were divided into a long-term chemotherapy subgroup (> 2 cycles) and a short-term chemotherapy subgroup (≤ 2 cycles). The results showed that the ORR of GP+BJOE decreased with a short-term cycle (Supplementary Table 3b), and the leukopenia advantage of GP+KAI (compared to other CHIs) was decreased (Supplementary Table 4c). For evaluation criteria, we found that it did not exhibit a significant influence on the endpoint of the main outcomes (Supplementary Table 3a, Table 4b and Table 5b). In clinical treatment, GP regimen chemotherapy is mainly used for lung squamous cell carcinoma. A subgroup analysis was conducted after dividing the included studies into a mainly squamous cell carcinoma subgroup and a mainly adenocarcinoma subgroup. The results were consistent, except for the decreased efficacy of GP+CKSI (Supplementary Table 3c).

A previous study 19 reported that some CHIs may not exhibit a clear effect on adverse reactions without supportive treatment. Therefore, we divided the included studies involving adverse reactions into a supportive treatment subgroup and a no supportive treatment subgroup. In the no supportive treatment subgroup, the results showed that the leukopenia advantage of GP+KAI (compared to the other CHIs) was decreased (Supplementary Table 4a). Also, GP+CKSI, and GP+KAI showed no statistical differences in nausea and vomiting compared with GP regimen chemotherapy alone (Supplementary Table 5a).

To test the sensitivity of this NMA, we respectively excluded high-risk studies and not recommended dose studies. In the end, The SUCRAs ranking of different treatments outcomes were almost same as in Table 4. Therefore, the results of this meta-analysis are robust (Table 5).

### Inconsistency and publication bias

**Figure 7:**
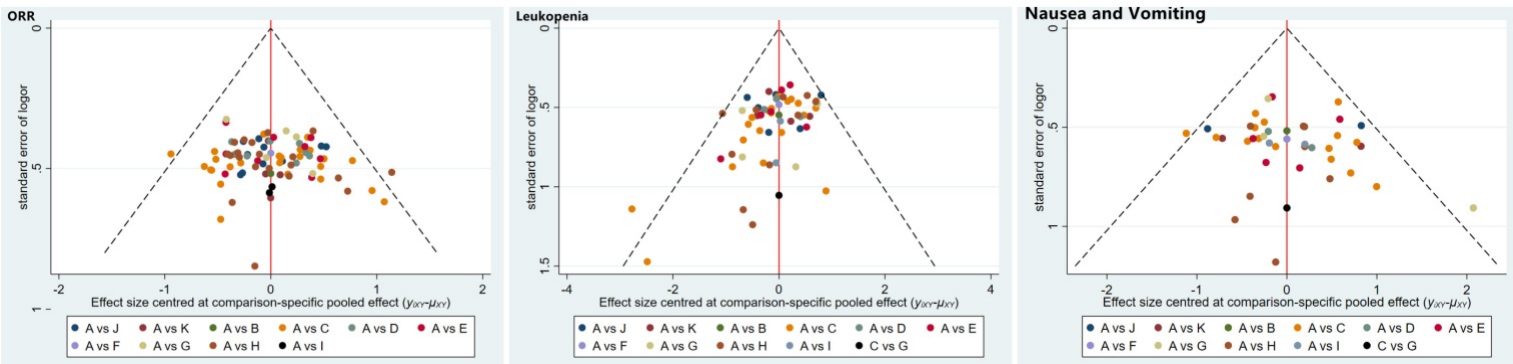
Funnel plots of the included randomized controlled trials.

**Figure 8:**
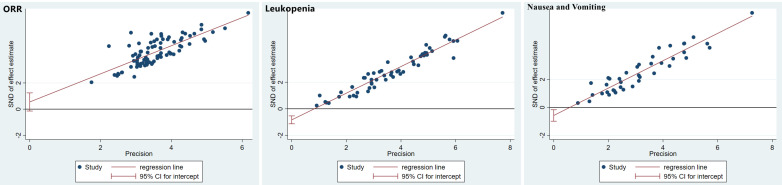
Egger's funnel plot with pseudo-95% confidence limits.

The publication bias was detected by qualitatively based on funnel-plot asymmetry (Figure 7) and quantitatively based on Egger's test (Figure 8). The points with different colors represent different comparisons between the interventions. We found possible publication bias, included nausea and vomiting (P=0.007), leukopenia (P=0.000). The Trim and Fill Method was used to further sensitivity analyses, the result showed there was no indications of publication with the Duval's trim and fill method (no new studies added).

## Discussion

NSCLC is a common respiratory malignancy worldwide. Although platinum-based chemotherapy has improved the clinical efficiency of NSCLC treatment, complementary therapy is still sought to reduce adverse reactions and improve the QOL of patients. Currently, TCM has been applied in multifaceted approaches and plays an indispensable role in the prevention and treatment of cancer, owing to its unique treatment concepts, theory, methods, and basic and clinical research 20. In clinical therapy, CHIs have become known for their rapid efficacy and convenient application characteristics compared to TCM decoctions. However, due to the large number of CHIs and the lack of direct control studies, it is difficult to select an optimal scheme of CHIs combined with GP regimen chemotherapy for the treatment of advanced NSCLC. Hence, we conducted a NMA to evaluate clinical efficiency, adverse reactions, QOL, and long-term synergistic efficacy in stage III/IV NSCLC patients treated with GP chemotherapy plus CHIs.

This study included 92 RCTs with 10 CHIs used in the treatment of NSCLC. Aidi, KLT, CKSI and KAI showed obvious advantages in both efficacy and safety, and among them, Aidi+GP (79.0%) showed great advantages of ORR, and KAI+GP and KLT+GP had the lowest probability in terms of leukopenia (4.4%) and nausea and vomiting (24.2%). KAI+GP might be the best performing combination. Based on the SUCRA analyses, this combination ranked first for reducing leukopenia, and was in the top three for improving ORR, reducing nausea and vomiting. Previous studies have demonstrated that KAI improves the effect and enhances QOL when used as an adjuvant treatment with chemotherapy. Such findings have been observed among patients with primary breast cancer 21, colorectal cancer 22, and NSCLC 23. Recent pharmacological studies have reported that flavonoids from Astragalus membranaceus (Fisch.) Bge. var. mongholicus (Bge.) Hsiao and rare ginsenosides are the major effective substances of KAI 24. It has also been reported that the flavonoid of Astragalus could inhibit the proliferation of K562 cells, and inhibit the tumor growth of C57 black (BL)/6 tumor-bearing mice by regulating immune 25,26. Furthermore, ginseng can inhibit tumor cell growth and differentiation, increase the sensitivity of chemotherapy drugs, and enhance the immune function of peripheral blood lymphocytes in patients with cancer 27. Besides, we also noticed anticancer drugs and adjuvant drugs for cancer got different advantages in the curative effects, such as high ORR of Aidi+GP. Through the cluster analysis, Aidi and CKSI showed better advantages than other anticancer drugs in ranking of efficient and safety in this study.

Differences in survival rates are of utmost importance to both clinicians and NSCLC patients. We are similarly interested in the long-term synergistic efficacy of CHIs compared with GP regimen chemotherapy alone. There were five CHIs (Aidi, KLT, CKSI, XAPI and ELMI) included in this NMA for which MST was reported, and KLT+GP was the only combination that showed a positive effect on MST compared with GP regimen chemotherapy alone.

GP regimen chemotherapy exhibits varying degrees of blood and gastrointestinal toxicity, and we selected the most common adverse clinical reactions, including nausea, vomiting, and leukopenia, to evaluate the role of CHIs in the prevention of adverse reactions. As the only CHI recommended for the treatment of leukopenia following chemotherapy 28, KAI also showed a clear advantage over other CHIs in this study. However, subgroup analyses found that, in the no supportive treatment subgroup, the leukopenia advantage of GP+KAI over other CHIs was decreased, and that GP+Aidi, GP+CKSI, and GP+KAI did not show statistical differences in nausea and vomiting compared with GP alone. The results of the subgroup analyses suggested that CHIs might have a limited effect in reducing adverse reactions. The results of the pathological pattern subgroup showed that CHIs might have a similar effect in squamous cell carcinoma and adenocarcinoma.

This study is the first to evaluate the efficacy and safety of different CHIs combined with GP regimen chemotherapy in the treatment of advanced NSCLC. Also, the rankings of CHIs according to their clinical effectiveness rates and other outcomes provides direction for clinical medication. However, there are some limitations of this study that should be noted. Firstly, each API+GP and ELMI+GP included only one study 106,109 on this study, which may limit the strength of the evidence. Secondly, some included trials were of low methodological quality. There were 43 studies reported the random allocation methods, but no study provided detailed information on the random allocation concealment. Additionally, all of the included RCTs were performed in China, which reduces the universal applicability of the results. Finally, a potential limitation was publication bias, due to the fact that studies obtaining optimistic results could be more easily published than studies with unfavorable results. Though the existence of publication bias, sensitivity analyses of Trim and Fill Method showed the result was reliable.

It should be noted that evidence concerning the long-term synergistic efficacy of different CHIs combined with GP regimen chemotherapy, such as overall survival and progression-free survival, is still insufficient. And most studies did not mention the stages of chemotherapy, which may affect the efficacy. Therefore, the studies with high methodological quality are still required. And we appeal to clinical researchers to clarify the details of chemotherapy, include short-term and long-term synergistic efficacy with the specific normative data type and regard it as a vital outcome in future research.

## Conclusions

In this network meta-analysis, KAI+GP of adjuvant drugs, Aidi+GP and CKSI+GP of anticancer drugs appeared to be the advantageous treatment options for patients with advanced NSCLC, owing to its superior therapeutic performance and reduced adverse reactions. Also, KLT+GP positively affected MST. However, many shortcomings in clinical trial methodology resulted in an inadequate assessment of clinical efficacy and safety. Therefore, direct and diverse multicenter comparisons between different CHIs are warranted to further confirm these results, and present an in-depth review in the near future.

## Supplementary Material

Supplementary figures and tables.

## Figures and Tables

**Table 1:** Characteristics of included studies

First author. year	NSCLC (Ⅲ-Ⅳ)				Inventions			Scale (A)	Scale (B)	Supportive treatment	Outcome	
	E/C	M/F	Age	Ad/Sq/Ot	Treatment		Control				Main outcomes	Secondary outcomes
					E	CHI (D/C)						
Chen B. 2014 18	49/52	78/23	27-74	42/47/12	GP+Aidi	50-100ml/2	GP	RECIST	WHO	Yes	①②③	④⑤
Fu LJ. 2012 19	35/35	Unclear	61-84	43/26/1	GP+Aidi	50ml/2	GP	WHO	Unclear	Yes	①②	
Gen KJ. 2020 20	45/45	61/29	44-79	30/50/9	GP+Aidi	50ml/4	GP	WHO	Unclear	No	①	
Guo X. 2020 21	51/51	58/44	43-75	Unclear	GP+Aidi	60ml/4	GP	Unclear	Unclear	Yes	②③	④
Huang WJ. 2017 22	39/40	46/33	49-70	27/25/27	GP+Aidi	60ml/3	GP	RECIST	WHO	No	①②	
Kuang XK. 2008 23	26/13	33/6	34-71	22/12/5	GP+Aidi	50ml/?	GP	WHO	WHO	Yes	①②	
Li J. 2016 24	47/47	52/42	40-70	53/34/7	GP+Aidi	50-100ml/4	GP	WHO	WHO	Yes	①②③	
Liu H. 2010 25	32/32	37/27	45-75	35/15/14	GP+Aidi	50ml/4	GP	WHO	Unclear	No	①③	
Liu HF. 2019 26	44/44	54/34	42-76	39/12/37	GP+Aidi	50ml/2	GP	RECIST	Unclear	No	①	
Li XY. 2015 27	20/20/20	24/16	45-74	25/13/2	GP+Aidi GP+KAI	50ml/2 50ml/2	GP	Unclear	WHO	No	②③	⑤
Liu YH. 2014 28	43/43	53/33	39-73	49/37/0	GP+Aidi	50ml/2	GP	WHO	WHO	Yes	①②③	
Ma M. 2017 29	42/42	55/29	44-75	44/9/31	GP+Aidi	50ml/4	GP	WHO	Unclear	Yes	①②	
Shen RR. 2021 30	30/30	40/20	34-81	29/31/0	GP+Aidi	60ml/4	GP	WHO	Unclear	No	①	
Song ZZ. 2009 31	30/30	36/24	53-76	Unclear	GP+Aidi	50ml/2	GP	WHO	WHO	Yes	①③	⑤
Su SJ. 2017 32	40/39	45/34	40-70	31/39/9	GP+Aidi	50ml/2	GP	RECIST	Unclear	Yes	①②③	
Sun GS. 2008 33	33/30	54/9	34-73	30/24/9	GP+Aidi	10ml/2	GP	WHO	Unclear	Yes	①	④
Sun JB. 2012 34	34/34	42/26	60-86	41/25/2	GP+Aidi	50ml/2	GP	RECIST	NCI-CTC3	Yes	①②③	⑤
Wang SD. 2015 35	42/40	61/21	39-67	Unclear	GP+Aidi	50ml/2	GP	RECIST	Unclear	No	①②③	
Wen HQ. 2014 36	45/45	64/26	61-81	64/23/3	GP+Aidi	50ml/2	GP	RECIST	NCI-CTC3	No	①③	⑤
Wen K. 2009 37	38/38	52/24	32-77	28/48/0	GP+Aidi	50ml/2	GP	WHO	WHO	Yes	①②③	⑤
Wu T. 2017 38	67/68	83/52	43-71	48/87	GP+Aidi	100ml/2	GP	WHO	Unclear	NO	①②	
Xu H. 2013 39	38/42	55/25	39-81	31/49/0	GP+Aidi	50ml/2	GP	WHO	WHO	No	①②	⑤
Xu Y. 2012 40	33/33	36/30	Unclear	Unclear	GP+Aidi	80ml/4	GP	RECIST	WHO	Yes	①②③	
Xu ZJ. 2020 41	40/40	53/27	49-72	57/23/0	GP+Aidi	50-100ml/2	GP	WHO	Unclear	Yes	①②	
Zhang L. 2009 42	32/31	44/19	31-79	29/27/7	GP+Aidi	80ml/2	GP	WHO	WHO	Yes	①②	
Zhang XC. 2016 43	25/25	Unclear	Unclear	Unclear	GP+Aidi	50ml/4	GP	RECIST	WHO	No	①	⑤
Zhao J. 2019 44	43/43	55/31	Unclear	39/43/4	GP+Aidi	50ml/2	GP	WHO	Unclear	No	①	④
Zhao S. 2015 45	43/43	58/28	43-79	36/47/3	GP+Aidi	100ml/1	GP	WHO	WHO	Yes	①②③	⑤
Zhou DM. 2018 46	58/58	63/53	41-70	Unclear	GP+Aidi	50ml/2	GP	RECIST	Unclear	No	①②③	
Bao H. 2019 47	31/31	38/24	39-72	Unclear	GP+KLT	200ml/2	GP	WHO	Unclear	No	①③	
Chen C. 2018 48	30/30	Unclear	35-65	Unclear	GP+KLT	200ml/1	GP	RECIST	Unclear	Yes	①	
Chen W. 2016 49	44/44	47/41	55-78	58/20/10	GP+KLT	?/4	GP	RECIST	Unclear	No	①	
Chen Y. 2018 50	51/51	59/43	57-79	58/28/16	GP+KLT	200ml/4	GP	WHO	Unclear	No	①②	
Guan XQ. 2009 51	12/12	11/12	36-72	16/8/0	GP+KLT	300ml/2	GP	WHO	NCI-CTC	Yes	①②③	④
Gui XM. 2020 52	60/60	60/51	32/74	74/25/21	GP+KLT	200ml/2	GP	RECIST	WHO	No	①②	
Huang ZB. 2010 53	35/35	44/26	59-78	27/36/7	GP+KLT	200ml/2	GP	WHO	WHO	No	①②	⑤
Li HY. 2017 54	41/41	43/39	55-75	18/56/8	GP+KLT	?/4	GP	RECIST	Unclear	No	①	
Liang SG. 2014 55	23/20	Unclear	60-75	32/16/0	GP+KLT	100ml/2	GP	WHO	Unclear	No	①	⑤
Liu F. 2019 56	63/63	79/47	50-77	67/47/12	GP+KLT	200ml/2	GP	RECIST	Unclear	Yes	①	④⑤
Liu JQ. 2011 57	35/35	44/26	59-74	27/41/2	GP+KLT	200ml/2	GP	RECIST	WHO	Yes	①②③	⑤
Liu Y. 2015 58	43/43	55/31	42-74	41/42/3	GP+KLT	200ml/4	GP	RECIST	SFDA	Yes	①	⑤
Long SG. 2017 59	42/40	52/30	47-70	39/22/21	GP+KLT	200ml/3	GP	RECIST	WHO	No	①	
Sun SQ. 2012 60	35/35	41/29	37-75	22/38/10	GP+KLT	200ml/4	GP	WHO	WHO	No	①	
Wang L. 2014 61	43/43	58/28	43-79	36/47/3	GP+KLT	200ml/1	GP	RECIST	WHO	Yes	①②③	⑤
Wang Y. 2017 62	36/36	32/40	Unclear	49/23/0	GP+KLT	60ml/4	GP	WHO	Unclear	No	①②③	⑤
Yan QH. 2018 63	49/49	63/35	38-76	51/47/0	GP+KLT	200ml/4	GP	RECIST	Unclear	Yes	①②	
Yao J. 2017 64	70/67	78/59	Unclear	62/66/9	GP+KLT	200ml/2	GP	RECIST	WHO	No	①②③	
Ye CY. 2019 65	40/40	54/26	55-74	40/35/5	GP+KLT	200ml/2	GP	RECIST	Unclear	No	①③	
Zhang MM. 2019 66	50/50	52/48	Unclear	0/100/0	GP+KLT	?/3	GP	RECIST	Unclear	No	①②③	④
Duan P. 2009 67	72/71	88/55	36-69	69/47/27	GP+CKSI	20ml/2	GP	WHO	WHO	Yes	①②③	
Fan QL. 2015 68	63/63	74/52	27-68	60/47/19	GP+CKSI	25ml/2	GP	WHO	WHO	No	①	
Fen Q. 2018 69	40/40	42/38	Unclear	9/61/10	GP+CKSI	15-20ml/2	GP	RECIST	WHO	No	①	
Gao LJ. 2019 70	30/30	32/28	25-70	26/34/0	GP+CKSI	20ml/1	GP	RECIST	CTCAE4	No	①③	⑤
Liu Y. 2009 71	44/40	52/32	42-76	31/53/0	GP+CKSI	25ml/2	GP	WHO	WHO	Yes	①②③	⑤
Lu WL. 2017 72	60/600060	68/52	50-75	48/41/31	GP+CKSI	20ml/4	GP	WHO	WHO	Yes	①②	⑤
Wang ZX. 2009 73	30/30	49/11	38-75	Unclear	GP+CKSI	30ml/2	GP	RECIST	WHO	Yes	①②③	⑤
Zhang MY. 2019 74	52/48	53/47	51-76	60/36/4	GP+CKSI	20ml/2	GP	RECIST	Unclear	No	①②	⑤
Zhou HY. 2011 75	40/40	42/38	34-76	46/32/2	GP+CKSI	30ml/3	GP	WHO	WHO	No	①②③	④
Chen HL. 2010 76	45/41	62/24	38-71	44/37/5	GP+BJOE	30-40ml/4	GP	WHO	WHO	No	①②③	⑤
Liu SR. 2019 77	49/49	51/47	45-88	34/34/30	GP+BJOE	30ml/2	GP	WHO	WHO	Yes	①	
Su BK. 2017 78	29/28	42/15	Unclear	36/20/1	GP+BJOE	30ml/2	GP	Unclear	WHO	Yes	③	⑤
Tian L. 2017 79	48/48	63/33	40-81	33/50/13	GP+BJOE	30ml/2	GP	WHO	Unclear	Yes	①	⑤
Wang JH. 2012 80	68/68	94/42	52-74	37/99/0	GP+BJOE	30ml/4	GP	WHO	WHO	Yes	①	⑤
Wang LC. 2015 81	40/40	52/28	35-75	34/32/14	GP+BJOE	20-30ml/2	GP	WHO	WHO	No	①②	
Wang YZ. 2021 82	39/39	52/26	Unclear	Unclear	GP+BJOE	40ml/2	GP	WHO	Unclear	Yes	①	
Ye HN. 2015 83	54/53	68/39	Unclear	52/34/21	GP+BJOE	30ml/4	GP	RECIST	WHO	Yes	①②	
Yu HW. 2020 84	42/42	38-84	39/45	Unclear	GP+BJOE	30ml/3	GP	RECIST	Unclear	No	①	
Zhang B. 2017 85	39/39	54/24	Unclear	48/23/7	GP+BJOE	40ml/2	GP	WHO	Unclear	Yes	①	
An AJ. 2014 86	49/48	52/45	39-76	44/41/12	GP+SQFZI	250ml/2	GP	RECIST	Unclear	No	①	⑤
He WJ. 2008 87	35/35	46/24	38-75	32/38/0	GP+SQFZI	250ml/2	GP	WHO	WHO	Yes	①②	⑤
He WX. 2021 88	48/48	58/38	56-78	44/52/0	GP+SQFZI	250ml/4	GP	WHO	Unclear	No	①②	
Lin CH. 2014 89	32/30	40/22	41-70	39/22/3	GP+SQFZI	250ml/2	GP	Unclear	WHO	No	①	⑤
Lou T. 2020 90	40/40	58/22	>60	44/27/9	GP+SQFZI	250ml/4	GP	WHO	Unclear	No	①②③	
Luo BP. 2018 91	48/48	61/35	33-63	30/56/10	GP+SQFZI	250ml/2	GP	WHO	Unclear	No	①②	
Wang YQ. 2010 92	39/37	48/28	Unclear	Unclear	GP+SQFZI	250ml/2	GP	WHO	WHO	No	①	⑤
Yao DJ. 2013 93	50/50	84/16	30-70	73/27/0	GP+SQFZI	250ml/2	GP	WHO	WHO	Yes	①②	⑤
Zhang LM. 2017 94	52/52	59/45	41-82	Unclear	GP+SQFZI	250ml/2	GP	WHO	Unclear	Yes	①	
Zou T. 2009 95	35/35	43/27	34-70	42/24/4	GP+SQFZI	250ml/2	GP	WHO	WHO	Yes	①②③	⑥
Dong H. 2019 96	56/56	72/40	Unclear	62/42/8	GP+KAI	40ml/2	GP	WHO	Unclear	No	①	
Ge CZ. 2011 97	32/32	47/17	65-80	13/49/2	GP+KAI	30ml/?	GP	WHO	Unclear	Yes	①	⑥
Jiang H. 2018 98	43/42	52/33	Unclear	28/52/5	GP+KAI	50ml/2	GP	RECIST	CTCAE4	No	①②③	
Lu YZ. 2017 99	73/73	83/63	Unclear	58/72/16	GP+KAI	60ml/3	GP	WHO	WHO	Yes	①②③	
Shang LQ. 2011 100	32/31	37/26	39-71	30/29/4	GP+KAI	50ml/4	GP	Unclear	WHO	Yes	②	
Tao HZ. 2020 101	82/82	86/78	Unclear	Unclear	GP+KAI	50ml/4	GP	Unclear	Unclear	No	①	
Hu XL. 2017 102	53/53	66/40	332-74	54/39/13	GP+XAPI	20ml/4	GP	RECIST	WHO	Yes	①②	④
Li QL. 2016 103	36/36	54/18	27-74	28/42/2	GP+XAPI	40-60ml/2	GP	RECIST	WHO	Yes	①②③	④⑤
Liu JR. 2016 104	30/30	31/29	40-79	0/60/0	GP+XAPI	40ml/2	GP	RECIST	Unclear	No	①②③	
Zhang FY. 2011 105	24/24	31/17	50-75	30/18/0	GP+XAPI	40-60ml/2	GP	WHO	NCL	No	①②	⑤
Qin ZQ. 2009 106	32/32	46/18	Unclear	36/26/2	GP+API	250mg/2	GP	WHO	WHO	Yes	①②③	⑤
Han L. 2012 107	25/26	29/22	Unclear	30/18/3	GP+LTNI	2mg/2	GP	WHO	WHO	Yes	①②③	
Li JJ. 2013 108	30/22	31/21	43-81	35/11/6	GP+LTNI	2mg/2	GP	WHO	WHO	No	①②③	⑤
Chu DJ. 2010 109	41/41	48/34	32-78	45/36/1	GP+ELMI	500mg/3	GP	RECIST	WHO	Yes	①②③	④

**Note:** NSCLC: non-small cell lung cancer; E/C: experimental group /control group; CHI (D/C): dose/cycles; Ad/Sq/Ot: adenocarcinoma/squamous carcinoma/other; GP: cisplatin and gemcitabine; Aidi: Aidi injection; KLT: Kanglaite injection; CKSI: Compound Kushen injection; BJOE: Brucea javanica Oil Emulsion injection; SQFZ: Shenqi Fuzheng injection; KAI: Kangai injection; XAPI: Xiaoaiping injection; API: Astragalus polysaccharide injection; LTNI: Lentinan injection; ELMI: Elemene injection; scale. A: evaluation criteria of tumor response; scale. B: evaluation criteria of adverse reactions; RECIST: response evaluation criteria in solid tumors; NCI-CTC: National Cancer Institute Common Toxicity Criteria; ①: ORR = CR+PR; ②: leukopenia; ③: nausea and vomiting; ④: median survival time; ⑤: Karnofsky performance status (KPS).

**Table 2:** Results of the network meta-analysis of KPS (upper right quadrant) and ORR (lower left quadrant)

GP+ELMI	GP+LTNI	GP+API	GP+XAPI	GP+KAI	GP+SQFZ	GP+BJOE	GP+CKSI	GP+KLT	GP+Aidi	GP
**GP+ELMI**	NA	NA	NA	NA	NA	NA	NA	NA	NA	NA
1.25 (0.38,4.07)	**GP+LTNI**	1.20 (0.23,6.42)	0.68 (0.15,2.97)	0.88 (0.21,3.64)	0.99 (0.28,3.46)	0.76 (0.22,2.69)	0.88 (0.25,3.10)	0.76 (0.22,2.62)	0.71 (0.21,2.45)	**0.29 (0.09,0.94)**
1.03 (0.27,3.92)	0.82 (0.23,3.00)	**GP+API**	0.56 (0.13,2.46)	0.73 (0.18,3.02)	0.82 (0.24,2.87)	0.63 (0.18,2.23)	0.73 (0.21,2.57)	0.63 (0.18,2.18)	0.59 (0.17,2.03)	**0.24 (0.07,0.78)**
1.12 (0.41,3.04)	0.90 (0.35,2.28)	1.09 (0.35,3.35)	**GP+XAPI**	1.30 (0.40,4.23)	1.46 (0.55,3.86)	1.13 (0.42,3.01)	1.30 (0.49,3.46)	1.12 (0.43,2.92)	1.05 (0.41,2.71)	0.42 (0.17,1.02)
0.72 (0.28,1.83)	0.57 (0.24,1.37)	0.70 (0.24,2.03)	0.64 (0.35,1.16)	**GP+KAI**	1.12 (0.47,2.71)	0.87 (0.36,2.11)	1.00 (0.41,2.43)	0.86 (0.36,2.05)	0.81 (0.36,1.85)	**0.33 (0.15,0.71)**
0.88 (0.35,2.20)	0.71 (0.30,1.64)	0.86 (0.30,2.45)	0.79 (0.45,1.38)	1.23 (0.79,1.92)	**GP+SQFZ**	0.77 (0.43,1.39)	0.89 (0.50,1.60)	0.76 (0.44,1.33)	0.72 (0.43,1.22)	**0.29 (0.19,0.44)**
0.79 (0.32,1.98)	0.63 (0.27,1.47)	0.77 (0.27,2.20)	0.71 (0.40,1.24)	1.10 (0.70,1.73)	0.89 (0.60,1.33)	**GP+BJOE**	1.16 (0.64,2.10)	0.99 (0.56,1.76)	0.94 (0.54,1.61)	**0.38 (0.24,0.58)**
0.76 (0.31,1.91)	0.61 (0.26,1.42)	0.74 (0.26,2.13)	0.68 (0.39,1.19)	1.07 (0.68,1.66)	0.87 (0.58,1.28)	0.97 (0.65,1.44)	**GP+CKSI**	0.86 (0.49,1.51)	0.81 (0.47,1.38)	**0.32 (0.21,0.49)**
0.81 (0.33,1.98)	0.65 (0.28,1.48)	0.79 (0.28,2.21)	0.72 (0.43,1.22)	1.13 (0.76,1.69)	0.92 (0.65,1.29)	1.03 (0.72,1.46)	1.06 (0.75,1.50)	**GP+KLT**	0.95 (0.57,1.57)	**0.38 (0.26,0.56)**
0.72 (0.29,1.75)	0.57 (0.25,1.30)	0.70 (0.25,1.96)	0.64 (0.38,1.08)	1.00 (0.68,1.48)	0.81 (0.59,1.13)	0.91 (0.65,1.27)	0.94 (0.67,1.31)	0.89 (0.68,1.16)	**GP+Aidi**	**0.40 (0.29,0.56)**
1.34 (0.56,3.22)	1.08 (0.49,2.39)	1.31 (0.47,3.60)	1.20 (0.74,1.95)	**1.88 (1.33,2.65)**	**1.52 (1.16,2.01)**	**1.70 (1.28,2.27)**	**1.76 (1.33,2.33)**	**1.66 (1.35,2.04)**	**1.87 (1.56,2.24)**	**GP**

**Note:** GP: cisplatin and gemcitabine; Aidi: Aidi injection; KLT: Kanglaite injection; CKSI: Compound Kushen injection; BJOE: Brucea javanica Oil Emulsion injection; SQFZ: Shenqi Fuzheng injection; KAI: Kangai injection; XAPI: Xiaoaiping injection; API: Astragalus polysaccharide injection.

**Table 3:** Results of the network meta-analysis of nausea and vomiting (upper right quadrant) and leukopenia (lower left quadrant)

GP+ELMI	GP+LTNI	GP+API	GP+XAPI	GP+KAI	GP+SQFZ	GP+BJOE	GP+CKSI	GP+KLT	GP+Aidi	GP
**GP+ELMI**	1.12 (0.25,5.02)	0.87 (0.16,4.56)	1.07 (0.24,4.77)	0.97 (0.24,3.84)	1.18 (0.28,5.01)	2.31 (0.53,10.20)	1.29 (0.35,4.80)	0.86 (0.23,3.16)	1.16 (0.33,4.03)	2.41 (0.72,8.11)
0.79 (0.21,3.00)	**GP+LTNI**	0.77 (0.18,3.27)	0.96 (0.27,3.32)	0.86 (0.29,2.61)	1.06 (0.32,3.45)	2.07 (0.60,7.09)	1.16 (0.42,3.20)	0.77 (0.28,2.10)	1.03 (0.41,2.64)	2.16 (0.89,5.23)
1.14 (0.27,4.79)	1.45 (0.35,6.08)	**GP+API**	1.24 (0.29,5.20)	1.12 (0.30,4.17)	1.37 (0.34,5.45)	2.68 (0.64,11.12)	1.49 (0.43,5.19)	0.99 (0.29,3.41)	1.34 (0.41,4.34)	2.79 (0.89,8.71)
0.48 (0.16,1.39)	0.61 (0.21,1.76)	0.42 (0.13,1.36)	**GP+XAPI**	0.90 (0.30,2.70)	1.11 (0.34,3.59)	2.17 (0.64,7.37)	1.21 (0.44,3.32)	0.80 (0.29,2.18)	1.08 (0.43,2.74)	2.26 (0.94,5.42)
1.82 (0.60,5.56)	2.31 (0.76,7.06)	1.59 (0.47,5.43)	**3.82 (1.76,8.28)**	**GP+KAI**	1.23 (0.44,3.42)	2.40 (0.81,7.05)	1.34 (0.58,3.07)	0.89 (0.39,2.01)	1.20 (0.58,2.46)	**2.50 (1.29,4.84)**
0.51 (0.18,1.41)	0.64 (0.23,1.79)	0.44 (0.14,1.39)	1.06 (0.56,2.00)	**0.28 (0.14,0.56)**	**GP+SQFZ**	1.95 (0.61,6.23)	1.09 (0.43,2.77)	0.72 (0.29,1.81)	0.98 (0.42,2.26)	2.04 (0.93,4.46)
0.42 (0.14,1.25)	0.53 (0.18,1.59)	0.36 (0.11,1.23)	0.87 (0.41,1.85)	**0.23 (0.10,0.52)**	0.82 (0.41,1.63)	**GP+BJOE**	0.56 (0.21,1.51)	**0.37 (0.14,0.99)**	0.50 (0.20,1.24)	1.04 (0.44,2.45)
0.62 (0.22,1.72)	0.78 (0.28,2.18)	0.54 (0.17,1.70)	1.30 (0.69,2.44)	**0.34 (0.17,0.69)**	1.22 (0.70,2.12)	1.49 (0.75,2.96)	**GP+CKSI**	0.66 (0.33,1.33)	0.89 (0.50,1.61)	**1.87 (1.13,3.09)**
0.51 (0.19,1.40)	0.65 (0.24,1.78)	0.45 (0.14,1.39)	1.07 (0.58,1.98)	**0.28 (0.14,0.56)**	1.01 (0.59,1.71)	1.23 (0.63,2.40)	0.83 (0.49,1.40)	**GP+KLT**	1.35 (0.76,2.39)	**2.82 (1.74,4.58)**
0.57 (0.21,1.53)	0.73 (0.27,1.94)	0.50 (0.17,1.51)	1.20 (0.68,2.10)	**0.31 (0.17,0.60)**	1.13 (0.71,1.80)	1.38 (0.74,2.56)	0.93 (0.58,1.47)	1.12 (0.72,1.74)	**GP+Aidi**	**2.09 (1.54,2.83)**
**0.23 (0.09,0.59)**	**0.29 (0.11,0.75)**	**0.20 (0.07,0.59)**	**0.48 (0.29,0.79)**	**0.13 (0.07,0.23)**	**0.45 (0.31,0.67)**	**0.55 (0.31,0.97)**	**0.37 (0.25,0.55)**	**0.45 (0.31,0.64)**	**0.40 (0.31,0.52)**	**GP**

**Note:** GP: cisplatin and gemcitabine; Aidi: Aidi injection; KLT: Kanglaite injection; CKSI: Compound Kushen injection; BJOE: Brucea javanica Oil Emulsion injection; SQFZ: Shenqi Fuzheng injection; KAI: Kangai injection; XAPI: Xiaoaiping injection; API: Astragalus polysaccharide injection.

**Table 4:** Surface under the cumulative ranking curve results of the outcomes

	ORR (%)	Leukopenia (%)	Nausea and vomiting (%)	KPS (%)
GP	11.5	99.8	93.1	0.7
GP+Aidi	79.0	52.2	48.3	37.7
GP+API	42.5	20.0	31.3	74.6
GP+BJOE	62.6	75.3	85.4	45.8
GP+CKSI	68.6	46.3	56.9	59.8
GP+ELMI	44.2	24.0	39.0	-
GP+KAI	76.4	4.4	35.8	60.0
GP+KLT	60.8	61.9	24.2	45.6
GP+LTNI	27.1	36.0	45.5	64.7
GP+SQFZ	48.5	63.2	49.5	71.0
GP+XAPI	28.8	67.0	41.0	40.0

**Table 5:** The sensitivity of this NMA

	ORR (%)	Leukopenia (%)	Nausea and vomiting (%)
**A. Exclusion of high-risk studies**			
GP	12.2	99.7	92.1
GP+Aidi	87.1	58.9	43.3
GP+API	41.7	19	32.7
GP+BJOE	63.2	74.9	85.3
GP+CKSI	69	44	53.5
GP+ELMI	43.1	20.8	38.7
GP+KAI	69.1	11.4	54.5
GP+KLT	63.1	61	23.5
GP+LTNI	24.9	33.9	43.6
GP+SQFZ	48.1	60.8	40.9
GP+XAPI	28.5	65.5	41.9
**B. Exclusion of not recommended doses studies**			
GP	12.5	99.7	91.3
GP+Aidi	81.3	52.5	49.1
GP+API	42.3	21.6	32.3
GP+BJOE	66.2	65.3	89.4
GP+CKSI	68.5	46.1	57.8
GP+ELMI	42.8	24.4	39.8
GP+KAI	76.9	4.3	35.4
GP+KLT	56.1	67.2	26.4
GP+LTNI	23.9	36.4	44.4
GP+SQFZ	49.9	64.5	41
GP+XAPI	29.6	68.2	43.2
