# Supplementary material for: Comparative efficacy of 10 Chinese herbal injections combined with GP regimen chemotherapy for patients with advanced NSCLC a systematic review and network meta-analysis
Source: J Cancer. 2022 Jan 1;13(2):465–80. doi: 10.7150/jca.66410 (PMC8771519; doi:10.7150/jca.66410)
Supplement: Supplementary file 1 — Supplementary figures and tables. [file jcav13p0465s1.pdf]

Supplementary materials

Supplementary Figure 1: Electronic search strategy for Pubmed.

History and Search Details

Download Delete

Search	Actions	Details	Query	Results	Time
#18	···	>	Search: (((#1) AND (#2)) AND (#3)) OR (((#1) AND (#2)) AND (#4)) OR (((#1) AND (#2)) AND (#5)) OR (((#1) AND (#2)) AND (#6)) OR (((#1) AND (#2)) AND (#7)) OR (((#1) AND (#2)) AND (#8)) OR (((#1) AND (#2)) AND (#9)) OR (((#1) AND (#2)) AND (#10)) OR (((#1) AND (#2)) AND (#11)) OR (((#1) AND (#2)) AND (#12)) OR (((#1) AND (#2)) AND (#13)) OR (((#1) AND (#2)) AND (#14)) OR (((#1) AND (#2)) AND (#15)) OR (((#1) AND (#2)) AND (#16)) OR (((#1) AND (#2)) AND (#17)) Sort by: Publication Date	41	05:36:18
#17	···	>	Search: lentinan OR lentinan injection Sort by: Publication Date	651	05:29:57
#16	···	>	Search: Elemene injection OR Elemene Sort by: Publication Date	740	05:29:34
#15	···	>	Search: Astragalus polysaccharide injection OR Astragalus polysaccharide Sort by: Publication Date	726	05:29:20
#14	···	>	Search: Ginseng Polysacchride Injection OR Ginseng Polysacchride - Spellcheck off Sort by: Publication Date	1	05:29:01
#13	···	>	Search: ginseng polysaccharide injection OR ginseng polysaccharide Sort by: Publication Date	772	05:29:01
#12	···	>	Search: ginseng polysaccharide injection OR ginseng polysaccharide OR renshen duotang Sort by: Publication Date	772	05:17:58
#11	···	>	Search: Xiaoaiping injection OR Xiaoaiping Sort by: Publication Date	39	05:17:46
#10	···	>	Search: Shenqi Fuzheng injection OR Shenqi Fuzheng OR Shenqi Sort by: Publication Date	232	05:17:33
#9	···	>	Search: Brucea javanica Oil Emulsion injection OR Brucea javanica Oil Emulsion OR YaDanZi OR Yadanzi Sort by: Publication Date	43	05:17:20
#8	···	>	Search: Kangai injection or Kangai Sort by: Publication Date	504	05:17:08
#7	···	>	Search: Compound Kushen injection or Compound Kushen Sort by: Publication Date	88	05:16:56
#6	···	>	Search: Huachansu Injection OR Huachansu Sort by: Publication Date	82	05:16:40
#5	···	>	Search: Toad Venom Injection OR Toad Venom OR Chansu Sort by: Publication Date	5,174	05:16:27
#4	···	>	Search: Kanglaite OR Kanglaite injection OR kanglaite OR coix seed oil OR Coicis Semen Oil OR Coix Lacrymajobi Seed Oil Sort by: Publication Date	119	05:16:13
#3	···	>	Search: Aidi injection OR AiDi OR Aidi Sort by: Publication Date	161	05:15:55
#2	···	>	Search: Lung Neoplasm$1 OR Lung cancer$1 OR Non small cell lung cancer$1 OR NSCLC OR SCLC OR Pulmonary neoplasm$1 OR Pulmonary cancer$1 OR Lung carcinoma$1 OR Pulmonary carcinoma$1 OR Lung tumo?r$1 OR Pulmonary tumo?r$1 Sort by: Publication Date	225,733	05:12:24
#1	···	>	Search: Gemcitabine OR cis-platinum complex OR cis-platinum OR cisplatin Sort by: Publication Date	95,639	05:12:12

Showing 1 to 18 of 18 entries

Supplementary Table 1: Risk of bias summary.

First author. year	Random sequence generation	Allocation concealment	Blinding		Incomplete outcome data	Selective reporting	Other bias
			participants and personnel	outcome assessment			
An AJ.2014	Simple randomization	Unclear	Unclear	Unclear	complete	Low risk	Unclear
Bao H.2019	Simple randomization	Unclear	Unclear	Unclear	complete	Low risk	Unclear
Chen B.2014	Patient's opinion	Unclear	Unclear	Unclear	complete	Low risk	Unclear
Chen C.2018	random draw	Unclear	Unclear	Unclear	complete	Low risk	Unclear
Chen HL.2010	Unclear	Unclear	Unclear	Unclear	complete	Low risk	Unclear
Chen W.2016	Simple randomization	Unclear	Unclear	Unclear	complete	Low risk	Unclear
Chen Y.2018	Simple randomization	Unclear	Unclear	Unclear	complete	Low risk	Unclear
Chu DJ.2010	Unclear	Unclear	Unclear	Unclear	complete	Low risk	Unclear
Dong H.2019	Unclear	Unclear	Unclear	Unclear	complete	Low risk	Unclear
Duan P.2009	Unclear	Unclear	Unclear	Unclear	complete	Low risk	Unclear
Fan QL.2015	Simple randomization	Unclear	Unclear	Unclear	complete	Low risk	Unclear
Fen Q.2018	Unclear	Unclear	Unclear	Unclear	complete	Low risk	Unclear
Fu LJ.2012	Medical record number	Unclear	Unclear	Unclear	complete	Low risk	Unclear
Gao LJ.2019	Simple randomization	Unclear	Unclear	Unclear	complete	Low risk	Unclear
Ge CZ.2011	Unclear	Unclear	Unclear	Unclear	complete	Low risk	Unclear
Gen KJ.2020	Simple randomization	Unclear	Unclear	Unclear	complete	Low risk	Unclear
Guan XQ.2009	Unclear	Unclear	Unclear	Unclear	complete	Low risk	Unclear

Gui XM.2020	Unclear	Unclear	Unclear	Unclear	complete	Low risk	Unclear
Guo X.2020	Simple randomization	Unclear	Unclear	Unclear	complete	Low risk	Unclear
Han L.2012	Unclear	Unclear	Unclear	Unclear	complete	Low risk	Unclear
He WJ.2008	Unclear	Unclear	Unclear	Unclear	complete	Low risk	Unclear
He WX.2021	Simple randomization	Unclear	Unclear	Unclear	complete	Low risk	Unclear
Hu XL.2017	Simple randomization	Unclear	Unclear	Unclear	complete	Low risk	Unclear
Huang WJ.2017	Simple randomization	Unclear	Unclear	Unclear	complete	Low risk	Unclear
Huang ZB.2010	Unclear	Unclear	Unclear	Unclear	complete	Low risk	Unclear
Jiang H.2018	Simple randomization	Unclear	Unclear	Unclear	complete	Low risk	Unclear
Kuang XK.2008	Patient's opinion	Unclear	Unclear	Unclear	complete	Low risk	Unclear
Liang SG.2014	Simple randomization	Unclear	Unclear	Unclear	incomplete	Low risk	Unclear
Li HY.2017	Simple randomization	Unclear	Unclear	Unclear	complete	Low risk	Unclear
Li J.2016	Unclear	Unclear	Unclear	Unclear	complete	Low risk	Unclear
Li JJ.2013	Unclear	Unclear	Unclear	Unclear	complete	Low risk	Unclear
Lin CH.2014	Simple randomization	Unclear	Unclear	Unclear	complete	Low risk	Unclear
Li QL.2016	random draw	Unclear	Unclear	Unclear	complete	Low risk	Unclear
Liu F.2019	Simple randomization	Unclear	Unclear	Unclear	complete	Low risk	Unclear
Liu H.2010	Unclear	Unclear	Unclear	Unclear	complete	Low risk	Unclear
Liu HF.2019	Simple randomization	Unclear	Unclear	Unclear	complete	Low risk	Unclear
Liu JQ.2011	Unclear	Unclear	Unclear	Unclear	complete	Low risk	Unclear
Liu JR.2016	Simple randomization	Unclear	Unclear	Unclear	complete	Low risk	Unclear
Liu SR.2019	Simple randomization	Unclear	Unclear	Unclear	complete	Low risk	Unclear
Liu Y .2015	Unclear	Unclear	Unclear	Unclear	complete	Low risk	Unclear
Liu Y.2009	Unclear	Unclear	Unclear	Unclear	complete	Low risk	Unclear

Liu YH.2014	Simple randomization	Unclear	Unclear	Unclear	complete	Low risk	Unclear
Li XY.2015	Simple randomization	Unclear	Unclear	Unclear	complete	Low risk	Unclear
Long SG.2017	Unclear	Unclear	Unclear	Unclear	complete	Low risk	Unclear
Lou T.2020	Simple randomization	Unclear	Unclear	Unclear	complete	Low risk	Unclear
Luo BP.2018	Unclear	Unclear	Unclear	Unclear	complete	Low risk	Unclear
Lu WL.2017	Unclear	Unclear	Unclear	Unclear	complete	Low risk	Unclear
Lu YZ.2017	Medical record number	Unclear	Unclear	Unclear	complete	Low risk	Unclear
Ma M.2017	Simple randomization	Unclear	Unclear	Unclear	complete	Low risk	Unclear
Qin ZQ.2009	Unclear	Unclear	Unclear	Unclear	complete	Low risk	Unclear
Shang LQ.2011	Unclear	Unclear	Unclear	Unclear	complete	Low risk	Unclear
Shen RR.2021	Simple randomization	Unclear	Unclear	Unclear	complete	Low risk	Unclear
Song ZZ.2009	Unclear	Unclear	Unclear	Unclear	complete	Low risk	Unclear
Su BK.2017	Simple randomization	Unclear	Unclear	Unclear	complete	Low risk	Unclear
Su GS.2008	Unclear	Unclear	Unclear	Unclear	complete	Low risk	Unclear
Sun JB.2012	Medical record number	Unclear	Unclear	Unclear	complete	Low risk	Unclear
Sun SQ.2012	Simple randomization	Unclear	Unclear	Unclear	complete	Low risk	Unclear
Su SJ.2017	Simple randomization	Unclear	Unclear	Unclear	complete	Low risk	Unclear
Tao H.2020	Unclear	Unclear	Unclear	Unclear	complete	Low risk	Unclear
Tian L.2017	Unclear	Unclear	Unclear	Unclear	complete	Low risk	Unclear
Wang JH.2012	Simple randomization	Unclear	Unclear	Unclear	complete	Low risk	Unclear
Wang L.2014	Unclear	Unclear	Unclear	Unclear	complete	Low risk	Unclear
Wang LC.2015	Simple randomization	Unclear	Unclear	Unclear	complete	Low risk	Unclear
Wang SD.2015	Simple randomization	Unclear	Unclear	Unclear	complete	Low risk	Unclear
Wang Y.2017	Unclear	Unclear	Yes	Unclear	complete	Low risk	Unclear
Wang YQ.2010	Unclear	Unclear	Unclear	Unclear	complete	Low risk	Unclear
Wang YZ.2021	Unclear	Unclear	Unclear	Unclear	complete	Low risk	Unclear

Wang ZX.2009	Unclear	Unclear	Unclear	Unclear	complete	Low risk	Unclear
Wen HQ.2014	Simple randomization	Unclear	Unclear	Unclear	complete	Low risk	Unclear
Wen K.2009	Unclear	Unclear	Unclear	Unclear	complete	Low risk	Unclear
Wu T.2017	Unclear	Unclear	Unclear	Unclear	complete	Low risk	Unclear
Xu H.2013	Unclear	Unclear	Unclear	Unclear	complete	Low risk	Unclear
Xu Y.2012	Unclear	Unclear	Unclear	Unclear	complete	Low risk	Unclear
Xu ZJ.2020	Simple randomization	Unclear	Unclear	Unclear	complete	Low risk	Unclear
Yan QH.2018	Simple randomization	Unclear	Unclear	Unclear	complete	Low risk	Unclear
Yao DJ.2013	Simple randomization	Unclear	Unclear	Unclear	complete	Low risk	Unclear
Yao J.2017	Unclear	Unclear	Unclear	Unclear	complete	Low risk	Unclear
Ye CY.2017	Simple randomization	Unclear	Unclear	Unclear	complete	Low risk	Unclear
Ye HN.2015	Simple randomization	Unclear	Unclear	Unclear	complete	Low risk	Unclear
Yu HW.2020	Unclear	Unclear	Unclear	Unclear	complete	Low risk	Unclear
Zhang B.2017	Unclear	Unclear	Unclear	Unclear	complete	Low risk	Unclear
Zhang FY.2011	Unclear	Unclear	Unclear	Unclear	complete	Low risk	Unclear
Zhang L.2009	Unclear	Unclear	Unclear	Unclear	complete	Low risk	Unclear
Zhang LM.2017	Unclear	Unclear	Unclear	Unclear	complete	Low risk	Unclear
Zhang MM.2019	Simple randomization	Unclear	Unclear	Unclear	complete	Low risk	Unclear
Zhang MY.2019	Simple randomization	Unclear	Unclear	Unclear	complete	Low risk	Unclear
Zhang XC.2016	Unclear	Unclear	Unclear	Unclear	complete	Low risk	Unclear
Zhao J.2019	Simple randomization	Unclear	Unclear	Unclear	complete	Low risk	Unclear
Zhao S.2015	Unclear	Unclear	Unclear	Unclear	complete	Low risk	Unclear
Zhou DM.2018	Unclear	Unclear	Unclear	Unclear	complete	Low risk	Unclear
Zhou HY.2011	Unclear	Unclear	Unclear	Unclear	complete	Low risk	Unclear
Zhou T.2009	Unclear	Unclear	Unclear	Unclear	complete	Low risk	Unclear

Supplementary Figure 2: The meta-analysis of ORR between two groups.

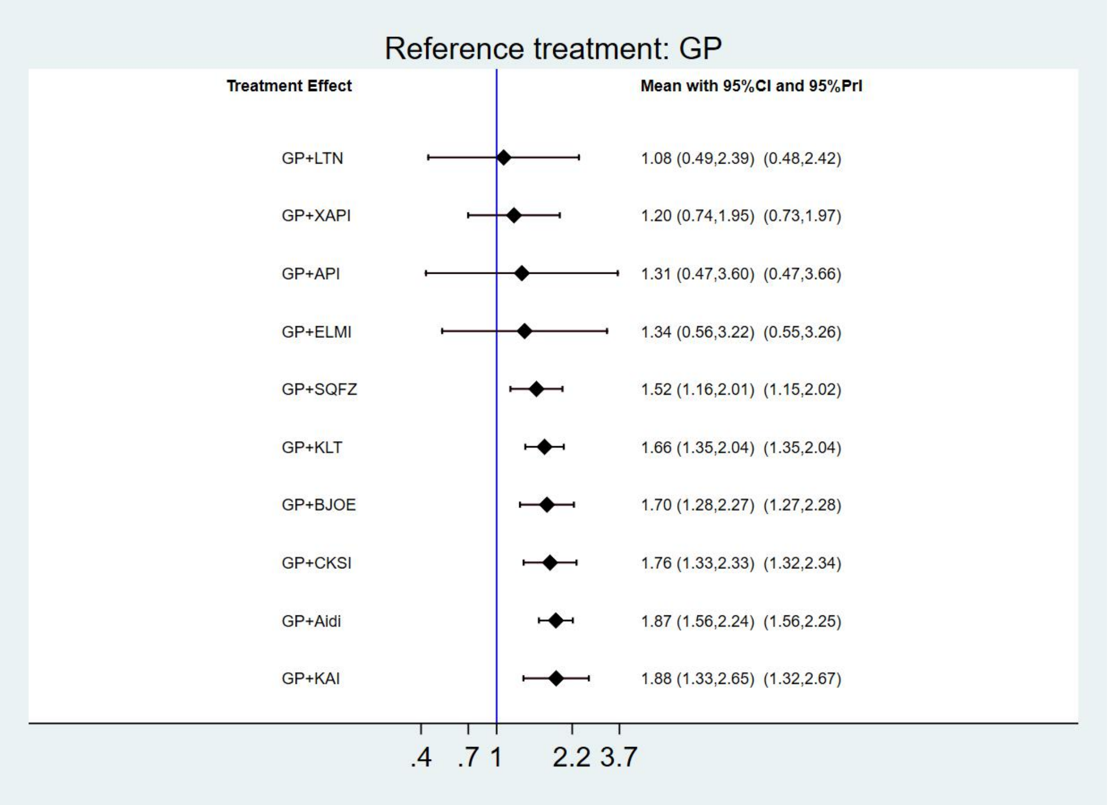

Supplementary Figure 3: The meta-analysis of leukopenia between two groups.

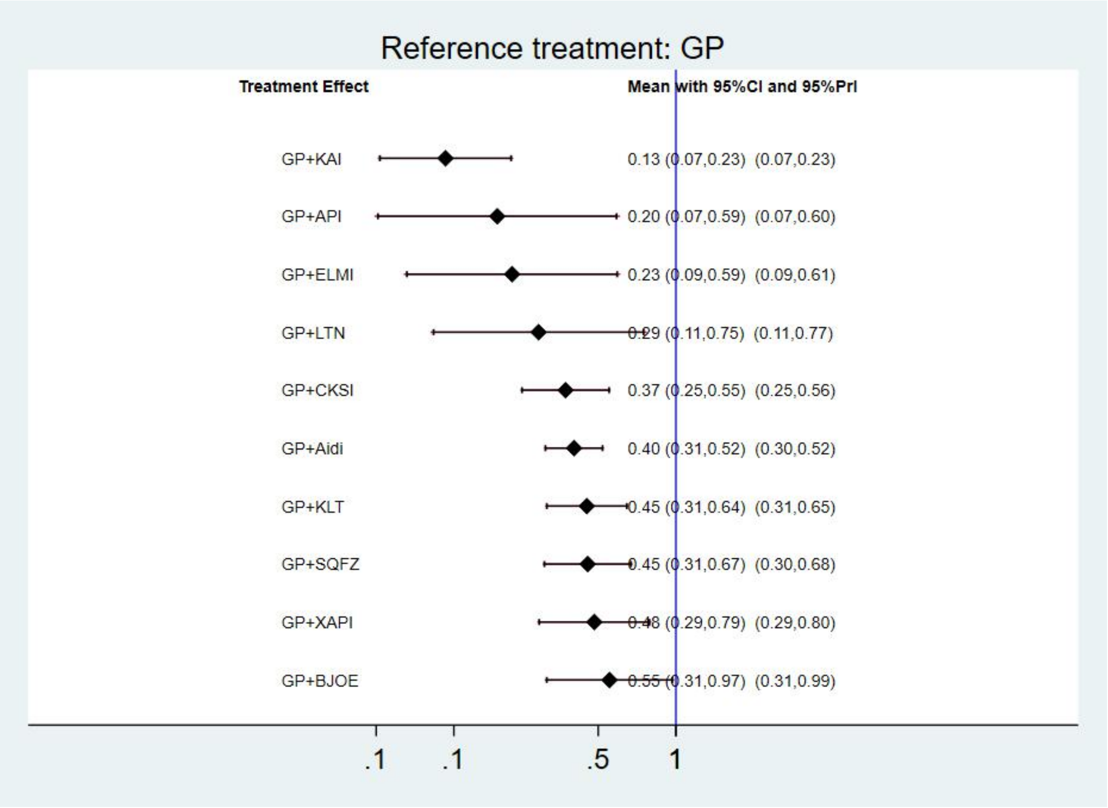

Supplementary Figure 4: The meta-analysis of nausea and vomiting between two groups.

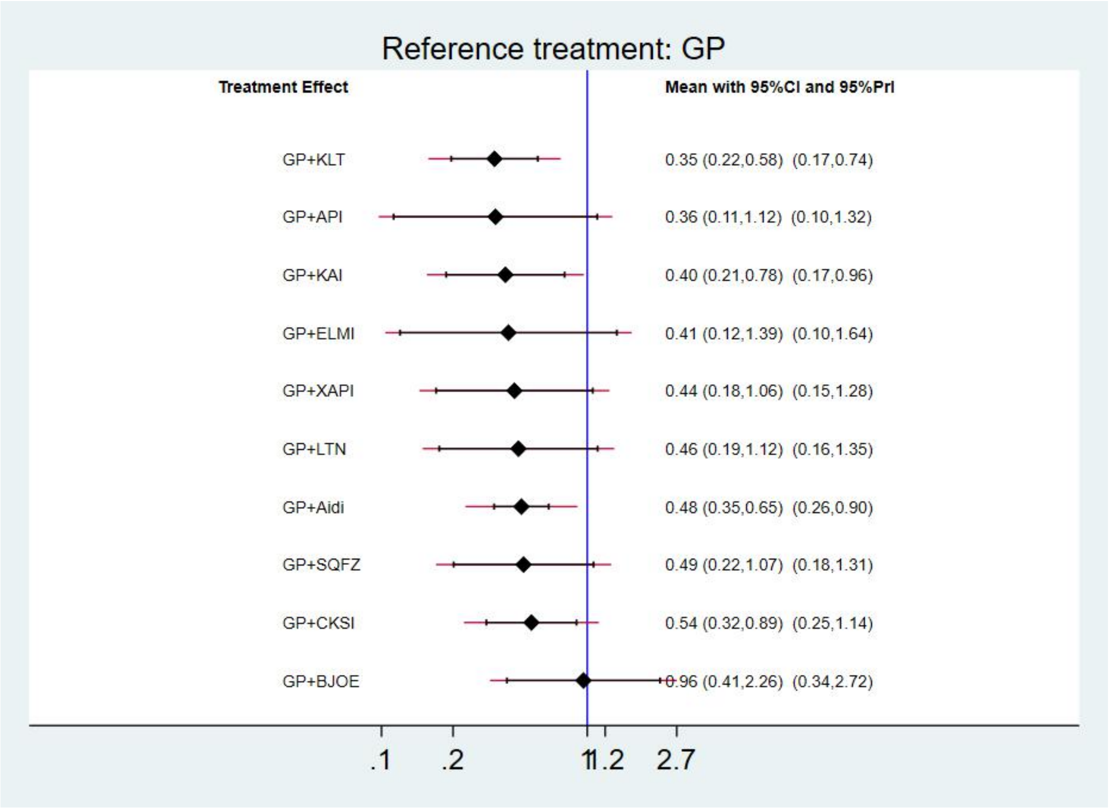

Supplementary Figure 5: Network graph of the KPS and MST.

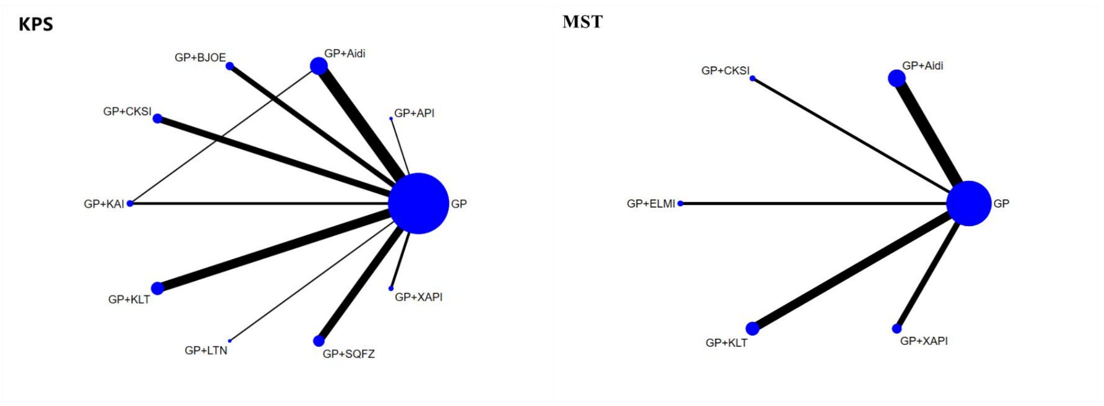

NOTE: Node sizes indicate the total sample sizes for treatments, and the line thickness corresponds to the number of trials.

Supplementary Figure 6: Results of the network meta-analysis of MST.

WinBUGS14 - [Node statistics]

File Tools Edit Attributes Info Model Inference Options Doodle Map Text W

node	mean	sd	MC error	2.5%	median	97.5%	start	sample
hr[2]	1.163	0.124	6.873E-4	0.9396	1.156	1.424	1	30000
hr[3]	1.458	0.1876	0.001101	1.124	1.447	1.859	1	30000
hr[4]	1.109	0.2491	0.001374	0.7038	1.082	1.669	1	30000
hr[5]	1.059	0.1589	9.861E-4	0.7798	1.047	1.402	1	30000
hr[6]	1.103	0.2462	0.001417	0.701	1.076	1.662	1	30000
rk[1,1]	0.2304	0.4211	0.002417	0.0	0.0	1.0	1	30000
rk[1,2]	0.4221	0.4939	0.002904	0.0	0.0	1.0	1	30000
rk[1,3]	0.2741	0.4461	0.002554	0.0	0.0	1.0	1	30000
rk[1,4]	0.06933	0.254	0.001465	0.0	0.0	1.0	1	30000
rk[1,5]	0.0041	0.0639	3.769E-4	0.0	0.0	0.0	1	30000
rk[1,6]	0.0	0.0	3.333E-13	0.0	0.0	0.0	1	30000
rk[2,1]	0.03093	0.1731	0.001026	0.0	0.0	1.0	1	30000
rk[2,2]	0.08597	0.2803	0.001581	0.0	0.0	1.0	1	30000
rk[2,3]	0.2031	0.4023	0.002437	0.0	0.0	1.0	1	30000
rk[2,4]	0.3295	0.47	0.002797	0.0	0.0	1.0	1	30000
rk[2,5]	0.3003	0.4584	0.002575	0.0	0.0	1.0	1	30000
rk[2,6]	0.05023	0.2184	0.00112	0.0	0.0	1.0	1	30000
rk[3,1]	5.333E-4	0.02309	1.387E-4	0.0	0.0	0.0	1	30000
rk[3,2]	0.0037	0.06071	3.544E-4	0.0	0.0	0.0	1	30000
rk[3,3]	0.018	0.133	7.857E-4	0.0	0.0	0.0	1	30000
rk[3,4]	0.05833	0.2344	0.001378	0.0	0.0	1.0	1	30000
rk[3,5]	0.2042	0.4031	0.002291	0.0	0.0	1.0	1	30000
rk[3,6]	0.7152	0.4513	0.002587	0.0	1.0	1.0	1	30000
rk[4,1]	0.2525	0.4345	0.002527	0.0	0.0	1.0	1	30000
rk[4,2]	0.1378	0.3447	0.002077	0.0	0.0	1.0	1	30000
rk[4,3]	0.1461	0.3532	0.002167	0.0	0.0	1.0	1	30000
rk[4,4]	0.1696	0.3753	0.002158	0.0	0.0	1.0	1	30000
rk[4,5]	0.1879	0.3906	0.002422	0.0	0.0	1.0	1	30000
rk[4,6]	0.1061	0.308	0.001786	0.0	0.0	1.0	1	30000
rk[5,1]	0.2253	0.4178	0.002475	0.0	0.0	1.0	1	30000
rk[5,2]	0.2067	0.4049	0.002358	0.0	0.0	1.0	1	30000
rk[5,3]	0.2139	0.4101	0.002413	0.0	0.0	1.0	1	30000
rk[5,4]	0.2033	0.4024	0.002548	0.0	0.0	1.0	1	30000
rk[5,5]	0.1225	0.3278	0.001855	0.0	0.0	1.0	1	30000
rk[5,6]	0.02847	0.1663	9.587E-4	0.0	0.0	1.0	1	30000
rk[6,1]	0.2602	0.4388	0.002656	0.0	0.0	1.0	1	30000
rk[6,2]	0.1439	0.351	0.001983	0.0	0.0	1.0	1	30000
rk[6,3]	0.1449	0.352	0.001936	0.0	0.0	1.0	1	30000
rk[6,4]	0.17	0.3756	0.002277	0.0	0.0	1.0	1	30000
rk[6,5]	0.1811	0.3851	0.00215	0.0	0.0	1.0	1	30000
rk[6,6]	0.09993	0.2999	0.001646	0.0	0.0	1.0	1	30000

NOTE: 1**:** cisplatin and gemcitabine; 2: Aidi injection plus cisplatin and gemcitabine; 3: Kanglaite injection plus cisplatin and gemcitabine; 4: Compound Kushen injection plus cisplatin and gemcitabine; 5: Xiaoaiping injection plus cisplatin and gemcitabine; 6: Elemene injection plus cisplatin and gemcitabine.

Supplementary Table 2: Node-splitting model resylts for outcomes.

A. **Node-splitting model resylts for leukopenia**

Treatment 1	Treatment 2	No. of studies	*P*
GP	Aidi	20	0.506
GP	KAI	4	0.250
Aidi	KAI	1	0.248

B. **Node-splitting model resylts for nausea and vomiting**

Treatment 1	Treatment 2	No. of studies	*P*
GP	Aidi	15	0.053
GP	KAI	3	0.937
Aidi	KAI	1	0.297

Note: GP: cisplatin and gemcitabine; Aidi: Aidi injection; KAI: Kangai injection.

Supplementary Table 3: Subgroup Analysis of ORR for Each Variable.

(a): Subgroup Analysis of ORR for RECIST evaluation criteria (upper right quadrant) and WHO evaluation criteria (lower left quadrant)

**GP+ELMI**	**GP+LTNI**	**GP+API**	**GP+XAPI**	**GP+KAI**	**GP+SQFZ**	**GP+BJOE**	**GP+CKSI**	**GP+KLT**	**GP+Aidi**	**GP**
**GP+ELMI**	NA	NA	0.89 (0.32,2.48)	1.34 (0.38,4.70)	1.83 (0.55,6.06)	1.48 (0.52,4.25)	1.39 (0.52,3.74)	1.15 (0.46,2.83)	1.42 (0.57,3.58)	0.74 (0.31,1.78)
NA	**GP+LTNI**	NA	NA	NA	NA	NA	NA	NA	NA	NA
NA	0.82 (0.23,3.00)	**GP+API**	NA	NA	NA	NA	NA	NA	NA	NA
NA	0.90 (0.22,3.74)	1.09 (0.23,5.18)	**GP+XAPI**	1.50 (0.53,4.27)	2.04 (0.77,5.44)	1.66 (0.75,3.67)	1.55 (0.76,3.16)	1.28 (0.72,2.30)	1.59 (0.87,2.93)	0.83 (0.49,1.42)
NA	0.45 (0.18,1.14)	0.55 (0.18,1.68)	0.51 (0.14,1.81)	**GP+KAI**	1.36 (0.40,4.63)	1.11 (0.38,3.26)	1.04 (0.37,2.87)	0.86 (0.34,2.18)	1.06 (0.41,2.75)	**0.56 (0.23,0.97)**
NA	0.74 (0.31,1.73)	0.89 (0.31,2.58)	0.82 (0.24,2.79)	1.62 (0.93,2.83)	**GP+SQFZ**	0.81 (0.29,2.24)	0.76 (0.29,1.96)	0.63 (0.27,1.48)	0.78 (0.33,1.87)	**0.41 (0.18,0.93)**
NA	0.66 (0.28,1.57)	0.80 (0.28,2.34)	0.74 (0.22,2.53)	1.46 (0.83,2.58)	0.90 (0.58,1.41)	**GP+BJOE**	0.94 (0.44,1.99)	0.77 (0.41,1.46)	0.96 (0.50,1.86)	**0.50 (0.28,0.91)**
NA	0.63 (0.26,1.51)	0.77 (0.26,2.24)	0.70 (0.20,2.42)	1.39 (0.78,2.49)	0.86 (0.54,1.36)	0.95 (0.59,1.53)	**GP+CKSI**	0.83 (0.49,1.40)	1.03 (0.59,1.79)	**0.54 (0.33,0.86)**
NA	0.53 (0.22,1.29)	0.64 (0.22,1.91)	0.59 (0.17,2.06)	1.17 (0.64,2.15)	0.72 (0.44,1.18)	0.80 (0.48,1.33)	0.84 (0.50,1.42)	**GP+KLT**	1.24 (0.85,1.81)	**0.65 (0.51,0.82)**
NA	0.58 (0.25,1.33)	0.71 (0.25,2.00)	0.65 (0.19,2.17)	1.28 (0.77,2.15)	0.79 (0.54,1.16)	0.88 (0.59,1.31)	0.92 (0.61,1.40)	1.10 (0.70,1.73)	**GP+Aidi**	**0.52 (0.39,0.70)**
NA	1.08 (0.49,2.39)	1.31 (0.47,3.60)	1.20 (0.37,3.92)	**2.37 (1.49,3.77)**	**1.46 (1.08,1.98)**	**1.62 (1.17,2.25)**	**1.70 (1.20,2.41)**	**2.03 (1.37,3.00)**	**1.85 (1.47,2.32)**	**GP**

(b): Subgroup Analysis of ORR for ≤ 2 cycle of chemotherapy (upper right quadrant) and > 2 cycle of chemotherapy (lower left quadrant)

GP+ELMI	GP+LTNI	GP+API	GP+XAPI	GP+KAI	GP+SQFZ	GP+BJOE	GP+CKSI	GP+KLT	GP+Aidi	GP
**GP+ELMI**	NA	NA	NA	NA	NA	NA	NA	NA	NA	NA
NA	**GP+LTNI**	1.21 (0.33,4.41)	1.13 (0.41,3.10)	1.97 (0.74,5.30)	1.35 (0.57,3.18)	1.29 (0.53,3.15)	1.45 (0.61,3.41)	1.61 (0.69,3.74)	1.60 (0.70,3.64)	0.93 (0.42,2.06)
NA	NA	**GP+API**	0.93 (0.28,3.06)	1.63 (0.51,5.24)	1.11 (0.39,3.22)	1.07 (0.36,3.17)	1.19 (0.41,3.45)	1.32 (0.46,3.79)	1.32 (0.47,3.71)	0.77 (0.28,2.11)
1.14 (0.35,3.71)	NA	NA	**GP+XAPI**	1.75 (0.75,4.07)	1.19 (0.60,2.38)	1.14 (0.55,2.37)	1.28 (0.64,2.56)	1.42 (0.72,2.79)	1.41 (0.74,2.71)	0.82 (0.44,1.52)
0.85 (0.31,2.29)	NA	NA	0.74 (0.30,1.86)	**GP+KAI**	0.68 (0.35,1.32)	0.66 (0.33,1.32)	0.73 (0.38,1.42)	0.81 (0.43,1.55)	0.81 (0.44,1.50)	**0.47 (0.26,0.84)**
0.74 (0.26,2.14)	NA	NA	0.65 (0.24,1.74)	0.87 (0.40,1.88)	**GP+SQFZ**	0.96 (0.58,1.58)	1.07 (0.69,1.67)	1.19 (0.78,1.80)	1.18 (0.81,1.72)	**0.69 (0.50,0.94)**
0.63 (0.24,1.65)	NA	NA	0.55 (0.23,1.34)	0.74 (0.39,1.39)	0.85 (0.41,1.77)	**GP+BJOE**	1.12 (0.67,1.85)	1.24 (0.77,2.00)	1.23 (0.79,1.93)	0.72 (0.49,1.06)
0.50 (0.18,1.44)	NA	NA	0.44 (0.17,1.17)	0.59 (0.28,1.26)	0.68 (0.29,1.58)	0.80 (0.39,1.65)	**GP+CKSI**	1.11 (0.73,1.70)	1.10 (0.75,1.62)	**0.64 (0.47,0.88)**
0.85 (0.34,2.13)	NA	NA	0.74 (0.32,1.72)	1.00 (0.57,1.75)	1.15 (0.58,2.25)	1.35 (0.81,2.25)	1.68 (0.87,3.25)	**GP+KLT**	0.99 (0.70,1.41)	**0.58 (0.44,0.76)**
0.56 (0.22,1.43)	NA	NA	0.49 (0.21,1.15)	0.66 (0.37,1.18)	0.76 (0.38,1.51)	0.89 (0.52,1.53)	1.11 (0.56,2.19)	0.66 (0.42,1.04)	**GP+Aidi**	**0.58 (0.47,0.72)**
1.34 (0.56,3.22)	NA	NA	1.17 (0.53,2.58)	**1.58 (1.05,2.55)**	**1.82 (1.01,3.32)**	**2.14 (1.41,3.25)**	**2.67 (1.49,4.79)**	**1.59 (1.18,2.14)**	**2.41 (1.71,3.39)**	**GP**

(c): Subgroup Analysis of ORR for squamous cell carcinomas ≤ adenocarcinoma (upper right quadrant) and squamous cell carcinomas > adenocarcinoma (lower left quadrant)

**GP+ELMI**	**GP+LTNI**	**GP+API**	**GP+XAPI**	**GP+KAI**	**GP+SQFZ**	**GP+BJOE**	**GP+CKSI**	**GP+KLT**	**GP+Aidi**	**GP**
**GP+ELMI**	NA	NA	NA	NA	NA	NA	NA	NA	NA	NA
1.25 (0.38,4.07)	**GP+LTNI**	NA	NA	NA	NA	NA	NA	NA	NA	NA
1.03 (0.27,3.92)	0.82 (0.23,3.00)	**GP+API**	NA	NA	NA	NA	NA	NA	NA	NA
1.14 (0.38,3.39)	0.91 (0.32,2.56)	1.10 (0.33,3.70)	**GP+XAPI**	1.65 (0.66,4.11)	1.44 (0.60,3.46)	1.80 (0.70,4.63)	1.30 (0.53,3.21)	1.35 (0.61,2.98)	1.57 (0.72,3.44)	0.82 (0.40,1.68)
0.56 (0.18,1.79)	0.45 (0.15,1.35)	0.55 (0.15,1.94)	0.49 (0.18,1.35)	**GP+KAI**	0.87 (0.41,1.85)	1.09 (0.48,2.50)	0.79 (0.36,1.72)	0.82 (0.43,1.57)	0.96 (0.51,1.81)	**0.50 (0.28,0.87)**
0.94 (0.36,2.43)	0.75 (0.31,1.82)	0.91 (0.31,2.70)	0.83 (0.39,1.76)	1.67 (0.72,3.90)	**GP+SQFZ**	1.25 (0.57,2.75)	0.90 (0.43,1.89)	0.94 (0.52,1.70)	1.09 (0.61,1.96)	**0.57 (0.34,0.93)**
0.89 (0.35,2.31)	0.71 (0.30,1.72)	0.87 (0.29,2.56)	0.78 (0.37,1.67)	1.59 (0.68,3.70)	0.95 (0.56,1.62)	**GP+BJOE**	0.72 (0.32,1.64)	0.75 (0.38,1.50)	0.88 (0.44,1.73)	**0.45 (0.25,0.84)**
0.70 (0.27,1.78)	0.56 (0.23,1.33)	0.68 (0.23,1.98)	0.61 (0.29,1.29)	1.24 (0.54,2.86)	0.74 (0.45,1.24)	0.78 (0.47,1.30)	**GP+CKSI**	1.04 (0.55,1.95)	1.21 (0.65,2.25)	0.63 (0.36,1.08)
0.85 (0.34,2.13)	0.68 (0.29,1.59)	0.83 (0.29,2.38)	0.75 (0.37,1.53)	1.52 (0.68,3.41)	0.91 (0.57,1.45)	0.96 (0.60,1.53)	1.22 (0.78,1.90)	**GP+KLT**	1.17 (0.75,1.81)	**0.60 (0.44,0.84)**
0.74 (0.30,1.83)	0.59 (0.25,1.37)	0.72 (0.25,2.04)	0.65 (0.32,1.31)	1.31 (0.59,2.93)	0.78 (0.50,1.24)	0.83 (0.52,1.31)	1.05 (0.68,1.63)	0.86 (0.59,1.27)	**GP+Aidi**	**0.52 (0.38,0.70)**
1.34 (0.56,3.22)	1.08 (0.49,2.39)	1.31 (0.47,3.60)	1.18 (0.61,2.28)	**2.39 (1.12,5.11)**	**1.43 (1.01,2.09)**	**1.51 (1.03,2.19)**	**1.92 (1.36,2.72)**	**1.57 (1.19,2.08)**	**1.82 (1.40,2.37)**	**GP**

Note: GP: cisplatin and gemcitabine; Aidi: Aidi injection; KLT: Kanglaite injection; CKSI: Compound Kushen injection; BJOE: Brucea javanica Oil Emulsion injection; SQFZ: Shenqi Fuzheng

injection; KAI: Kangai injection; XAPI: Xiaoaiping injection; API: Astragalus polysaccharide injection; LTNI: Lentinan injection; ELMI: Elemene injection.

Supplementary Table 4: Subgroup Analysis of leukopenia for Each Variable.

(a): Subgroup Analysis of leukopenia for no supportive treatment (upper right quadrant) and supportive treatment (lower left quadrant)

**GP+ELMI**	**GP+LTNI**	**GP+API**	**GP+XAPI**	**GP+KAI**	**GP+SQFZ**	**GP+BJOE**	**GP+CKSI**	**GP+KLT**	**GP+Aidi**	**GP**
**GP+ELMI**	NA	NA	NA	NA	NA	NA	NA	NA	NA	NA
0.77<br>(0.17,3.40)	**GP+LTNI**	NA	1.56<br>(0.25,9.82)	0.89<br>(0.14,5.66)	2.38<br>(0.42,13.65)	2.07<br>(0.34,12.78)	1.53<br>(0.24,9.65)	1.59<br>(0.28,8.95)	1.88<br>(0.34,10.46)	3.64<br>(0.69,19.19)
1.14<br>(0.27,4.78)	1.49<br>(0.31,7.17)	**GP+API**	NA	NA	NA	NA	NA	NA	NA	NA
0.44<br>(0.14,1.38)	0.58<br>(0.16,2.15)	0.39<br>(0.11,1.35)	**GP+XAPI**	0.57<br>(0.18,1.77)	1.53<br>(0.59,3.95)	1.33<br>(0.45,3.92)	0.98<br>(0.32,3.00)	1.02<br>(0.41,2.54)	1.21<br>(0.50,2.94)	**2.33**<br>**(1.06,5.13)**
3.76<br>(1.05,13.49)	4.90<br>(1.17,20.57)	3.29<br>(0.83,13.02)	**8.49**<br>**(2.92,24.71)**	**GP+KAI**	2.68<br>(1.02,7.06)	2.33<br>(0.78,6.99)	1.72<br>(0.56,5.34)	1.79<br>(0.71,4.55)	2.12<br>(0.86,5.21)	**4.09**<br>**(1.82,9.21)**
0.79<br>(0.26,2.39)	1.03<br>(0.28,3.72)	0.69<br>(0.20,2.34)	1.78<br>(0.76,4.21)	**0.21**<br>**(0.07,0.59)**	**GP+SQFZ**	0.87<br>(0.35,2.15)	0.64<br>(0.25,1.66)	0.67<br>(0.33,1.34)	0.79<br>(0.40,1.55)	1.53<br>(0.90,2.59)
0.44<br>(0.12,1.58)	0.57<br>(0.13,2.41)	0.38<br>(0.10,1.52)	0.98<br>(0.33,2.90)	**0.12**<br>**(0.03,0.39)**	0.55<br>(0.19,1.57)	**GP+BJOE**	0.74<br>(0.25,2.18)	0.77<br>(0.32,1.84)	0.91<br>(0.39,2.12)	1.76<br>(0.84,3.68)
0.64<br>(0.23,1.83)	0.84<br>(0.24,2.88)	0.56<br>(0.18,1.80)	1.45<br>(0.67,3.16)	**0.17**<br>**(0.06,0.45)**	0.81<br>(0.39,1.69)	1.47<br>(0.55,3.93)	**GP+CKSI**	1.04<br>(0.42,2.59)	1.23<br>(0.51,3.00)	**2.38**<br>**(1.08,5.23)**
0.49<br>(0.16,1.48)	0.64<br>(0.18,2.31)	0.43<br>(0.13,1.45)	1.11<br>(0.48,2.60)	**0.13**<br>**(0.05,0.37)**	0.62<br>(0.28,1.40)	1.13<br>(0.40,3.19)	0.77<br>(0.37,1.57)	**GP+KLT**	1.18<br>(0.64,2.19)	**2.28**<br>**(1.44,3.61)**
0.66<br>(0.24,1.80)	0.86<br>(0.26,2.85)	0.58<br>(0.19,1.78)	1.50<br>(0.73,3.05)	**0.18**<br>**(0.07,0.44)**	0.84<br>(0.43,1.62)	1.52<br>(0.60,3.85)	1.03<br>(0.60,1.79)	1.35<br>(0.70,2.57)	**GP+Aidi**	**1.93**<br>**(1.28,2.92)**
**0.23**<br>**(0.09,0.59)**	**0.30**<br>**(0.09,0.94)**	**0.20**<br>**(0.07,0.59)**	**0.52**<br>**(0.27,0.97)**	**0.06**<br>**(0.03,0.14)**	**0.29**<br>**(0.16,0.52)**	0.52<br>(0.22,1.25)	**0.36**<br>**(0.23,0.56)**	**0.46**<br>**(0.26,0.82)**	**0.34**<br>**(0.25,0.47)**	**GP**

(b): Subgroup Analysis of leukopenia for no evaluation criteria (upper right quadrant) and evaluation criteria (lower left quadrant)

**GP+ELMI**	**GP+LTNI**	**GP+API**	**GP+XAPI**	**GP+KAI**	**GP+SQFZ**	**GP+BJOE**	**GP+CKSI**	**GP+KLT**	**GP+Aidi**	**GP**
**GP+ELMI**	NA	NA	NA	NA	NA	NA	NA	NA	NA	NA
0.79 (0.21,3.00)	**GP+LTNI**	NA	NA	NA	NA	NA	NA	NA	NA	NA
1.14 (0.27,4.78)	1.45 (0.35,6.07)	**GP+API**	NA	NA	NA	NA	NA	NA	NA	NA
0.43 (0.14,1.28)	0.54 (0.18,1.63)	0.37 (0.11,1.25)	**GP+XAPI**	NA	3.14 (0.81,12.26)	NA	1.96 (0.38,10.01)	2.19 (0.65,7.40)	1.45 (0.46,4.57)	**3.14 (1.07,9.27)**
1.83 (0.60,5.57)	2.32 (0.76,7.07)	1.60 (0.47,5.44)	**4.27 (1.90,9.61)**	**GP+KAI**	NA	NA	NA	NA	NA	NA
0.63 (0.22,1.80)	0.81 (0.28,2.29)	0.56 (0.17,1.77)	1.48 (0.73,3.02)	**0.35 (0.17,0.73)**	**GP+SQFZ**	NA	0.62 (0.14,2.72)	0.70 (0.26,1.89)	0.46 (0.18,1.15)	1.00 (0.44,2.29)
0.42 (0.14,1.25)	0.53 (0.18,1.59)	0.36 (0.11,1.22)	0.97 (0.44,2.15)	**0.23 (0.10,0.51)**	0.65 (0.32,1.34)	**GP+BJOE**	NA	NA	NA	NA
0.65 (0.23,1.84)	0.83 (0.30,2.33)	0.57 (0.18,1.81)	1.53 (0.77,3.06)	**0.36 (0.17,0.73)**	1.03 (0.56,1.88)	1.58 (0.78,3.16)	**GP+CKSI**	1.12 (0.29,4.29)	0.74 (0.20,2.67)	1.60 (0.47,5.45)
0.69 (0.24,1.97)	0.87 (0.31,2.50)	0.60 (0.19,1.94)	1.61 (0.78,3.31)	**0.38 (0.18,0.80)**	1.08 (0.57,2.05)	1.66 (0.80,3.43)	1.05 (0.57,1.95)	**GP+KLT**	0.66 (0.33,1.31)	1.43 (0.82,2.51)
0.63 (0.23,1.72)	0.80 (0.30,2.19)	0.55 (0.18,1.70)	1.48 (0.77,2.83)	**0.35 (0.18,0.68)**	1.00 (0.57,1.73)	1.52 (0.79,2.93)	0.97 (0.57,1.64)	0.92 (0.52,1.62)	**GP+Aidi**	**2.17 (1.47,3.21)**
**0.23 (0.09,0.59)**	**0.29 (0.11,0.75)**	**0.20 (0.07,0.59)**	**0.53 (0.31,0.93)**	**0.13 (0.07,0.23)**	**0.36 (0.23,0.56)**	**0.55 (0.31,0.97)**	**0.35 (0.23,0.53)**	**0.33 (0.21,0.53)**	**0.36 (0.26,0.50)**	**GP**

(c): Subgroup Analysis of leukopenia for ≤ 2 cycle of chemotherapy (upper right quadrant) and > 2 cycle of chemotherapy (lower left quadrant)

**GP+ELMI**	**GP+LTNI**	**GP+API**	**GP+XAPI**	**GP+KAI**	**GP+SQFZ**	**GP+BJOE**	**GP+CKSI**	**GP+KLT**	**GP+Aidi**	**GP**
**GP+ELMI**	NA	NA	NA	NA	NA	NA	NA	NA	NA	NA
NA	**GP+LTNI**	0.69 (0.16,2.94)	1.87 (0.59,5.95)	0.83 (0.23,2.92)	1.49 (0.51,4.36)	1.44 (0.35,5.82)	1.29 (0.43,3.82)	1.14 (0.39,3.31)	1.32 (0.48,3.62)	**3.45 (1.32,8.97)**
NA	NA	**GP+API**	2.72 (0.76,9.66)	1.20 (0.31,4.70)	2.17 (0.66,7.15)	2.08 (0.47,9.29)	1.87 (0.56,6.26)	1.66 (0.50,5.43)	1.92 (0.62,5.97)	**5.00 (1.68,14.88)**
0.58 (0.17,1.97)	NA	NA	**GP+XAPI**	0.44 (0.15,1.26)	0.80 (0.35,1.79)	0.77 (0.23,2.57)	0.69 (0.30,1.58)	0.61 (0.27,1.36)	0.71 (0.34,1.45)	1.84 (0.96,3.52)
3.76 (1.05,13.49)	NA	NA	6.53 (2.04,20.87)	**GP+KAI**	1.81 (0.69,4.71)	1.74 (0.47,6.47)	1.56 (0.59,4.14)	1.38 (0.53,3.57)	1.60 (0.67,3.85)	**4.17 (1.83,9.53)**
0.47 (0.15,1.50)	NA	NA	0.81 (0.29,2.29)	**0.12 (0.04,0.37)**	**GP+SQFZ**	0.96 (0.31,2.98)	0.86 (0.42,1.75)	0.76 (0.39,1.50)	0.89 (0.50,1.57)	**2.31 (1.42,3.75)**
0.37 (0.11,1.17)	NA	NA	0.63 (0.22,1.79)	**0.10 (0.03,0.29)**	0.78 (0.30,2.06)	**GP+BJOE**	0.90 (0.28,2.83)	0.79 (0.26,2.45)	0.92 (0.32,2.69)	2.40 (0.86,6.68)
0.63 (0.20,1.95)	NA	NA	1.10 (0.40,2.96)	**0.17 (0.06,0.48)**	1.35 (0.54,3.40)	1.72 (0.69,4.32)	**GP+CKSI**	0.89 (0.44,1.78)	1.03 (0.56,1.88)	**2.68 (1.59,4.51)**
0.33 (0.11,0.98)	NA	NA	0.57 (0.22,1.49)	**0.09 (0.03,0.24)**	0.70 (0.29,1.70)	0.90 (0.37,2.16)	0.52 (0.23,1.19)	**GP+KLT**	1.16 (0.66,2.04)	**3.02 (1.89,4.83)**
0.46 (0.16,1.32)	NA	NA	0.80 (0.32,1.98)	**0.12 (0.05,0.32)**	0.99 (0.43,2.26)	1.26 (0.55,2.87)	0.73 (0.34,1.57)	1.40 (0.68,2.90)	**GP+Aidi**	**2.60 (1.91,3.55)**
**0.23 (0.09,0.59)**	NA	NA	**0.40 (0.18,0.87)**	**0.06 (0.03,0.14)**	**0.49 (0.25,0.97)**	0.62 (0.32,1.24)	**0.36 (0.20,0.67)**	0.70 (0.40,1.22)	**0.50 (0.31,0.79)**	**GP**

Note: GP: cisplatin and gemcitabine; Aidi: Aidi injection; KLT: Kanglaite injection; CKSI: Compound Kushen injection; BJOE: Brucea javanica Oil Emulsion injection; SQFZ: Shenqi Fuzheng injection; KAI: Kangai injection; XAPI: Xiaoaiping injection; API: Astragalus polysaccharide injection; LTNI: Lentinan injection; ELMI: Elemene injection.

Supplementary Table 5: Subgroup Analysis of nausea and vomiting for Each Variable.

(a): Subgroup Analysis of nausea and vomiting for no supportive treatment (upper right quadrant) and supportive treatment (lower left quadrant)

**GP+ELMI**	**GP+LTNI**	**GP+API**	**GP+XAPI**	**GP+KAI**	**GP+SQFZ**	**GP+BJOE**	**GP+CKSI**	**GP+KLT**	**GP+Aidi**	**GP**
**GP+ELMI**	NA	NA	NA	NA	NA	NA	NA	NA	NA	NA
0.73<br>(0.15,3.58)	**GP+LTNI**	NA	0.56<br>(0.08,3.93)	1.57<br>(0.27,9.25)	0.54<br>(0.08,3.56)	2.03<br>(0.30,13.70)	1.02<br>(0.18,5.77)	0.88<br>(0.19,4.20)	1.27<br>(0.28,5.78)	2.63<br>(0.65,10.57)
1.16<br>(0.26,5.15)	1.58<br>(0.34,7.30)	**GP+API**	NA	NA	NA	NA	NA	NA	NA	NA
0.41<br>(0.08,2.06)	0.57<br>(0.11,2.91)	0.36<br>(0.08,1.68)	**GP+XAPI**	2.79<br>(0.49,15.97)	0.95<br>(0.15,6.17)	3.62<br>(0.55,23.70)	1.82<br>(0.31,10.69)	1.57<br>(0.34,7.22)	2.26<br>(0.51,9.94)	4.67<br>(0.98,18.11)
1.35<br>(0.37,4.95)	1.84<br>(0.48,7.06)	1.17<br>(0.34,4.00)	3.26<br>(0.84,12.69)	**GP+KAI**	0.34<br>(0.06,1.85)	1.30<br>(0.24,7.12)	0.63<br>(0.13,3.00)	0.56<br>(0.15,2.07)	0.81<br>(0.24,2.72)	1.67<br>(0.56,5.02)
0.37<br>(0.09,1.58)	0.50<br>(0.11,2.24)	0.32<br>(0.08,1.29)	0.89<br>(0.20,4.03)	0.27<br>(0.08,0.89)	**GP+SQFZ**	3.79<br>(0.61,23.54)	1.91<br>(0.34,10.58)	1.65<br>(0.38,7.08)	2.37<br>(0.58,9.72)	**4.89**<br>**(1.36,17.61)**
0.33<br>(0.07,1.65)	0.45<br>(0.09,2.34)	0.29<br>(0.06,1.35)	0.80<br>(0.15,4.19)	0.24<br>(0.06,0.96)	0.90<br>(0.20,4.12)	**GP+BJOE**	0.50<br>(0.09,2.84)	0.43<br>(0.10,1.90)	0.63<br>(0.15,2.62)	1.29<br>(0.35,4.74)
0.69<br>(0.21,2.32)	0.95<br>(0.27,3.32)	0.60<br>(0.19,1.86)	1.67<br>(0.47,5.97)	0.51<br>(0.22,1.21)	1.89<br>(0.64,5.60)	2.10<br>(0.58,7.60)	**GP+CKSI**	0.86<br>(0.25,2.98)	1.24<br>(0.38,4.06)	2.56<br>(0.92,7.13)
1.06<br>(0.29,3.89)	1.45<br>(0.38,5.55)	0.92<br>(0.27,3.14)	2.56<br>(0.66,9.97)	0.79<br>(0.29,2.10)	2.89<br>(0.88,9.48)	3.22<br>(0.82,12.69)	1.53<br>(0.65,3.62)	**GP+KLT**	1.44<br>(0.58,3.59)	**2.97**<br>**(1.48,5.95)**
0.89<br>(0.28,2.80)	1.21<br>(0.37,4.01)	0.77<br>(0.26,2.24)	2.14<br>(0.64,7.23)	0.66<br>(0.30,1.43)	2.42<br>(0.87,6.71)	2.69<br>(0.79,9.21)	1.28<br>(0.70,2.35)	0.84<br>(0.39,1.81)	**GP+Aidi**	**2.06**<br>**(1.14,3.74)**
0.41<br>(0.14,1.24)	0.57<br>(0.18,1.78)	**0.36**<br>**(0.13,0.99)**	1.00<br>(0.31,3.21)	**0.31**<br>**(0.15,0.62)**	1.13<br>(0.43,2.96)	1.26<br>(0.39,4.10)	**0.60**<br>**(0.36,0.99)**	**0.39**<br>**(0.19,0.78)**	**0.47**<br>**(0.33,0.66)**	**GP**

(b): Subgroup Analysis of nausea and vomiting for no evaluation criteria (upper right quadrant) and evaluation criteria (lower left quadrant)

GP+ELMI	GP+LTNI	GP+API	GP+XAPI	GP+KAI	GP+SQFZ	GP+BJOE	GP+CKSI	GP+KLT	GP+Aidi	GP
**GP+ELMI**	NA	NA	NA	NA	NA	NA	NA	NA	NA	NA
0.89<br>(0.23,3.49)	**GP+LTNI**	NA	NA	NA	NA	NA	NA	NA	NA	NA
1.16<br>(0.26,5.15)	1.29<br>(0.35,4.73)	**GP+API**	NA	NA	NA	NA	NA	NA	NA	NA
0.41<br>(0.08,2.06)	0.46<br>(0.11,1.91)	0.36<br>(0.08,1.68)	**GP+XAPI**	NA	0.95<br>(0.22,4.17)	NA	NA	1.38<br>(0.38,5.02)	2.97<br>(0.91,9.72)	4.67<br>(0.98,13.87)
1.11<br>(0.33,3.78)	1.24<br>(0.47,3.29)	0.96<br>(0.30,3.04)	2.68<br>(0.74,9.73)	**GP+KAI**	NA	NA	NA	NA	NA	NA
0.37<br>(0.09,1.58)	0.41<br>(0.12,1.44)	0.32<br>(0.08,1.29)	0.89<br>(0.20,4.03)	0.33<br>(0.11,1.00)	**GP+SQFZ**	NA	NA	1.44<br>(0.43,4.86)	3.12<br>(1.04,9.33)	**4.89<br>(1.81,13.21)**
0.43<br>(0.11,1.66)	0.49<br>(0.16,1.49)	0.38<br>(0.11,1.35)	1.05<br>(0.26,4.25)	0.39<br>(0.15,1.01)	1.18<br>(0.34,4.07)	**GP+BJOE**	NA	NA	NA	NA
0.78<br>(0.24,2.52)	0.87<br>(0.35,2.17)	0.67<br>(0.22,2.02)	1.87<br>(0.54,6.51)	0.70<br>(0.35,1.41)	2.11<br>(0.74,6.08)	1.79<br>(0.74,4.33)	**GP+CKSI**	NA	NA	NA
1.03<br>(0.30,3.53)	1.15<br>(0.43,3.08)	0.89<br>(0.28,2.84)	2.48<br>(0.68,9.08)	0.93<br>(0.42,2.04)	2.80<br>(0.92,8.56)	2.37<br>(0.91,6.16)	1.32<br>(0.65,2.70)	**GP+KLT**	2.16<br>(0.94,4.98)	**3.39<br>(1.69,6.79)**
1.00<br>(0.32,3.14)	1.12<br>(0.47,2.67)	0.86<br>(0.30,2.51)	2.41<br>(0.72,8.12)	0.90<br>(0.48,1.69)	2.72<br>(0.98,7.54)	2.30<br>(0.99,5.33)	1.29<br>(0.74,2.22)	0.97<br>(0.50,1.87)	**GP+Aidi**	1.57<br>(0.99,2.50)
0.41<br>(0.14,1.24)	0.46<br>(0.21,1.04)	**0.36<br>(0.13,0.99)**	1.00<br>(0.31,3.21)	**0.37<br>(0.22,0.65)**	1.13<br>(0.43,2.96)	0.95<br>(0.44,2.06)	**0.53<br>(0.35,0.82)**	**0.40<br>(0.23,0.71)**	**0.41<br>(0.30,0.58)**	**GP**

(c): Subgroup Analysis of nausea and vomiting for ≤ 2 cycle of chemotherapy (upper right quadrant) and > 2 cycle of chemotherapy (lower left quadrant)

**GP+ELMI**	**GP+LTNI**	**GP+API**	**GP+XAPI**	**GP+KAI**	**GP+SQFZ**	**GP+BJOE**	**GP+CKSI**	**GP+KLT**	**GP+Aidi**	**GP**
**GP+ELMI**	NA	NA	NA	NA	NA	NA	NA	NA	NA	NA
NA	**GP+LTNI**	0.77<br>(0.19,3.21)	0.95<br>(0.28,3.27)	1.23<br>(0.33,4.58)	2.43<br>(0.61,9.74)	2.71<br>(0.58,12.74)	1.25<br>(0.45,3.51)	0.73<br>(0.26,2.02)	1.24<br>(0.48,3.19)	2.16<br>(0.90,5.18)
NA	NA	**GP+API**	1.23<br>(0.30,5.09)	1.59<br>(0.36,7.07)	3.15<br>(0.67,14.90)	3.51<br>(0.64,19.17)	1.62<br>(0.46,5.63)	0.95<br>(0.28,3.25)	1.60<br>(0.49,5.20)	2.79<br>(0.91,8.57)
NA	NA	NA	**GP+XAPI**	1.29<br>(0.35,4.75)	2.55<br>(0.64,10.14)	2.84<br>(0.61,13.27)	1.31<br>(0.47,3.64)	0.77<br>(0.28,2.10)	1.30<br>(0.51,3.31)	2.26<br>(0.95,5.37)
1.35<br>(0.37,4.95)	NA	NA	NA	**GP+KAI**	1.97<br>(0.46,8.45)	2.20<br>(0.44,10.97)	1.01<br>(0.33,3.09)	0.59<br>(0.20,1.80)	1.00<br>(0.36,2.79)	1.75<br>(0.66,4.65)
2.03<br>(0.46,8.90)	NA	NA	NA	1.50<br>(0.45,5.05)	**GP+SQFZ**	1.11<br>(0.21,5.91)	0.51<br>(0.15,1.71)	0.30<br>(0.09,0.99)	0.51<br>(0.16,1.58)	0.89<br>(0.30,2.60)
0.53<br>(0.12,2.39)	NA	NA	NA	0.40<br>(0.12,1.36)	0.26<br>(0.06,1.10)	**GP+BJOE**	0.46<br>(0.12,1.84)	0.27<br>(0.07,1.07)	0.46<br>(0.12,1.72)	0.80<br>(0.22,2.85)
1.13<br>(0.24,5.31)	NA	NA	NA	0.84<br>(0.23,3.05)	0.56<br>(0.13,2.44)	2.11<br>(0.47,9.42)	**GP+CKSI**	0.58<br>(0.28,1.24)	0.99<br>(0.52,1.90)	**1.73**<br>**(1.00,2.97)**
0.86<br>(0.16,4.54)	NA	NA	NA	0.64<br>(0.15,2.67)	0.42<br>(0.09,2.10)	1.61<br>(0.32,8.09)	0.76<br>(0.14,4.01)	**GP+KLT**	1.69<br>(0.91,3.17)	**2.95**<br>**(1.76,4.94)**
1.33<br>(0.40,4.44)	NA	NA	NA	0.98<br>(0.42,2.32)	0.66<br>(0.21,2.00)	2.48<br>(0.79,7.75)	1.17<br>(0.35,3.91)	1.54<br>(0.40,5.95)	**GP+Aidi**	**1.74**<br>**(1.22,2.49)**
0.41<br>(0.14,1.24)	NA	NA	NA	**0.31**<br>**(0.15,0.62)**	**0.20**<br>**(0.08,0.55)**	0.77<br>(0.28,2.15)	0.37<br>(0.12,1.09)	0.48<br>(0.14,1.68)	**0.31**<br>**(0.19,0.52)**	**GP**

Note: GP: cisplatin and gemcitabine; Aidi: Aidi injection; KLT: Kanglaite injection; CKSI: Compound Kushen injection; BJOE: Brucea javanica Oil Emulsion injection; SQFZ: Shenqi Fuzheng injection; KAI: Kangai injection; XAPI: Xiaoaiping injection; API: Astragalus polysaccharide injection; LTNI: Lentinan injection; ELMI: Elemene injection.
